# XR SERIOUS GAMES FOR PHANTOM LIMB PAIN, NEUROFUNCTIONAL RECOVERY, AND PROSTHETIC ADOPTION: A SYSTEMATIC REVIEW

**DOI:** 10.2340/jrm.v58.46784

**Published:** 2026-11-13

**Authors:** Muhannad FARHAN, Saad Jawaid KHAN, Kehinde Quasim YUSUF, Abu Zeeshan BARI, Muhammad Faisal SIDDIQUI, Maram Ali ALAHMADI, Joyce Zhanzi WANG

**Affiliations:** 1 Department of Prosthetics and Orthotics, College of Medical Rehabilitation Sciences, Taibah University, Madinah, 42353, Saudi Arabia; 2 Department of Engineering Technology, Sam Houston State University, Huntsville, TX, USA; 3 Department of Computer Engineering, College of Computer Science and Information Technology, King Faisal University, Al-Ahsa 31982, Kingdom of Saudi Arabia; 4 Prosthetics and Orthotics Department, The Northern Medical Tower, Ministry of Health, Arar, Saudi Arabia; 5 EPIC Lab, Kids Research, The Children’s Hospital at Westmead; Faculty of Medicine and Health, The University of Sydney, Sydney, Australia

**Keywords:** phantom limb pain, extended reality, serious games, neurofunctional recovery, embodiment, prosthetics rehabilitation, virtual reality, augmented reality, mixed reality

## Abstract

**Purpose:**

To synthesize evidence on Extended Reality (XR) serious games for Phantom Limb Pain (PLP), neurofunctional recovery, embodiment, and adoption following amputation.

**Materials and Methods:**

PubMed, Scopus, Web of Science, and IEEE Xplore were searched up to January 2026. Eligible studies included adults with limb amputation who received XR serious game interventions and reported rehabilitation or user-experience outcomes. Quality was assessed using the Mixed Methods Appraisal Tool (MMAT). Evidence certainty was rated using GRADE across 5 outcome domains.

**Results:**

24 studies met the inclusion criteria. XR interventions showed consistent PLP reduction, with AR-based phantom motor execution systems reporting the largest effect sizes (*d* = 0.9–1.3) and preliminary evidence of reversal of cortical reorganization. VR exergames improved gait and balance in 2 RCTs. Immersive VR and EMG systems indicated improved object manipulation, performance time, and motor control. Neurofunctional and embodiment outcomes provided mechanistically plausible evidence regarding cortical activity, embodiment, and physiological adaptation.

**Conclusions:**

XR serious games show preliminary benefit in reducing phantom limb pain, improving functional mobility, supporting prosthesis control, and enhancing embodiment after amputation. Evidence linking XR-mediated sensorimotor feedback to cortical adaptation remains exploratory but is supported by neurofunctional findings. Usability and engagement outcomes were positive, although real-world implementation and scalability remain under-researched.

## LAY ABSTRACT

Many people who lose a limb continue to feel pain in the missing part of their body, known as phantom limb pain. This review looked at whether “extended reality” technologies, like virtual reality games and headsets that mix real and computer-generated views, can help. We combined results from 24 studies involving adults who had lost a limb. The findings were encouraging. Games that let people “move” their phantom limb using motion-sensing technology reduced pain noticeably. Virtual reality exercise games improved balance and walking. Other systems helped people better control artificial limbs and improve hand movements. These tools were also well liked and easy to use. While more research is needed before these games become standard treatment, this review suggests they could offer a low-risk, engaging way to ease pain and support recovery after limb loss, complementing, not replacing, conventional rehabilitation.

Limb amputation causes major disruption to sensorimotor congruence, body image, and independence. A common complication is phantom limb pain (PLP), experienced by 60–80% of amputees at some point in their lives (1, 2). Recent research indicates that PLP may involve not only peripheral nociception but also pathological cortical reorganization and changes in sensorimotor congruence and body schema (3–5). Despite the range of therapeutic interventions, such as pharmacotherapy, neuromodulation, and psychological interventions, results have proven variable, and long-term relief is limited (6, 7).

Traditionally, mirror therapy has sought to re-establish visual-motor congruence and re-calibrate the cortex (8, 9). However, logistical limitations such as limited posture and range of motion, and limited illusion stability, have prompted investigation of immersive digital solutions (7). Extended reality (XR), including virtual reality (VR), augmented reality (AR), and mixed reality, offers immersive, interactive, and multisensory environments that can increase engagement and potentially modulate pain (10–12).

Recent XR reviews have largely centred on pain reduction in PLP (7, 13, 14). Meanwhile, VR serious games have been explored for training in prosthetic control, motor training and motor learning, and device usability (15, 16). These studies show that immersive, interactive settings can support engagement and learning and provide realistic simulations for measuring performance and workload. Yet these 2 bodies of research have developed in parallel: XR-for-PLP research prioritizes pain reduction, while VR prosthetic research prioritizes functional measures of task performance, workload, and prosthesis usability. We still lack an integrated understanding of the link between pain reduction, neurofunctional rehabilitation, embodiment, and long-term acceptance of prostheses.

These data suggest VR serious games may affect rehabilitation outcomes not only through distraction and entertainment, but through recovery of integrated sensorimotor function. If embodiment is contingent upon appropriate temporal and kinematic information and congruent feedback, then serious game design becomes a neurofunctional factor (17–20).

The objective of this review is to critically explore the impact of XR serious games on phantom limb pain, neurofunctional recovery, embodiment, implementation, and adoption of prostheses in real-world settings following amputation. By placing XR interventions in the context of interrelated mechanistic and translational levels, we aim to clarify how XR interventions work, where evidence is lacking, and to guide future research designs that assess pain reduction, as well as other outcomes.

To guide this analysis and position the review in relation to prior XR and PLP literature, we propose a multi-level conceptual framework (Fig. 1). It depicts a layered hierarchy in which neurofunctional mechanisms (mirror principles, motor imagery, electromyography (EMG)-based interaction, multisensory feedback) drive the user-experience layer (enjoyment, embodiment, agency), which in turn governs implementation outcomes (feasibility, adherence, usability) and ultimately clinical adoption and pain relief. Embodiment acts as an intermediary between neuroplastic adaptation and prosthesis integration, with feedback loops linking functional benefit to pain modulation.

**Fig. 1:**
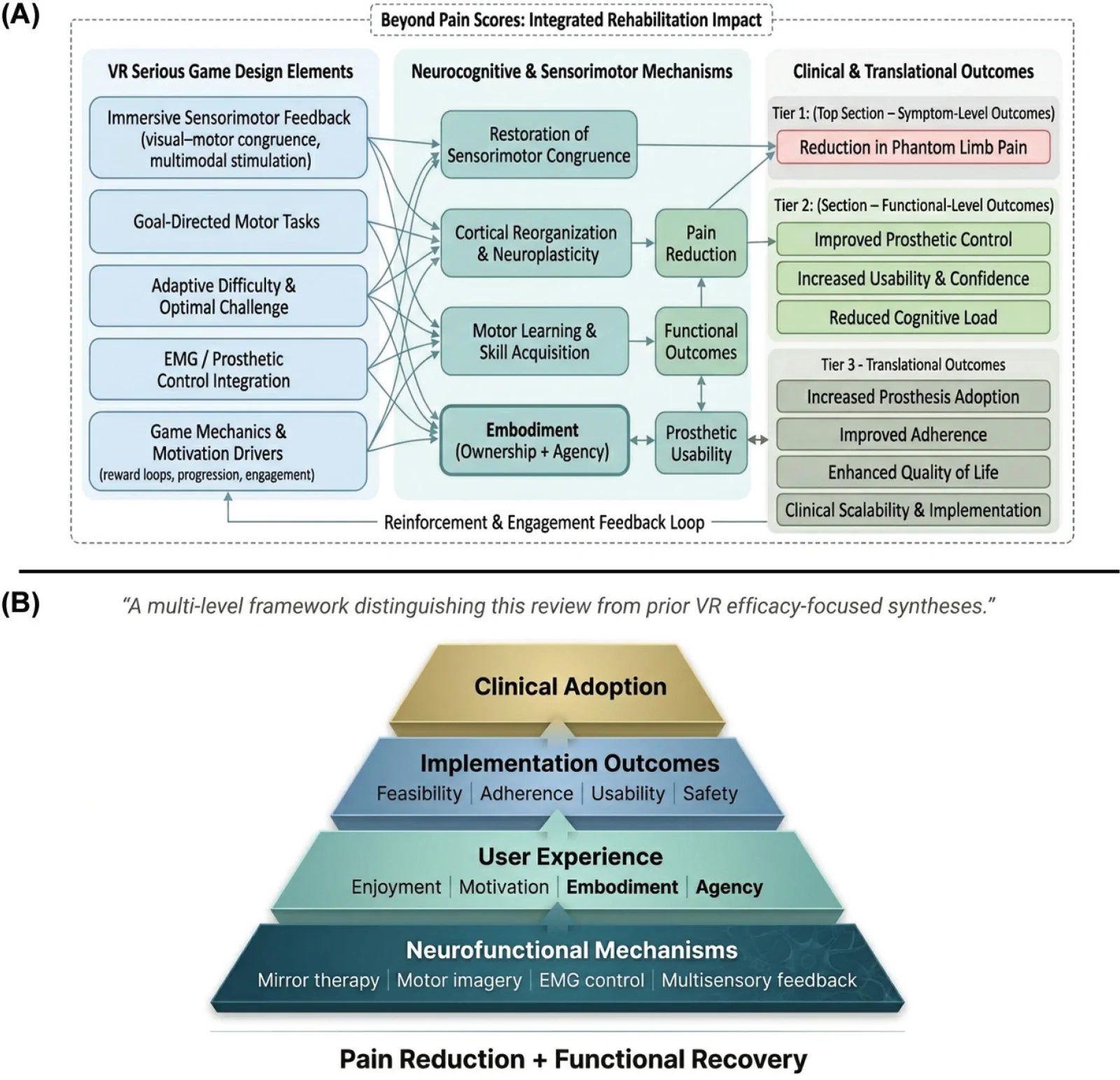
(A) Conceptual framework illustrating how virtual reality serious games may influence phantom limb pain, neurofunctional recovery, embodiment, and real-world prosthesis adoption. (B) Multi-level framework of XR-based rehabilitation. Neurofunctional mechanisms underpin user experience, which influences implementation outcomes and ultimately supports clinical adoption, leading to pain reduction and functional recovery.

In this conceptual framework, embodiment (ownership and agency) acts as an intermediary variable between the neuroplastic adaptation and prosthesis usability and integration. Functional benefits such as better control of the prosthesis and diminished cognitive effort (16) may play an indirect role in pain management and create feedback loops. The model also extends to prosthesis acceptance, adherence, quality of life, and system scalability.

## Methods

### Study design and reporting guidelines

The systematic review was conducted in accordance with the Preferred Reporting Items for Systematic Reviews and Meta-Analyses (PRISMA 2020) (21) guidelines to ensure rigorous and transparent reporting.

### Search strategy

We conducted a comprehensive search of electronic databases for studies on XR-based serious games for rehabilitation. Table SI provides a full list of Boolean search strings. The literature search was conducted between December 2025 and January 2026 in 4 electronic databases – PubMed, Scopus, Web of Science, and IEEE Xplore – from their inception to the latest search date.

Bibliometric analyses of the included studies were conducted via online tools such as Connected Papers (22) and SciSpace (23) by inputting the core papers. After duplicate removal, 2 reviewers independently screened titles and abstracts. Potentially relevant full texts were then retrieved and assessed. Disagreements were resolved by discussion and by consulting a third reviewer.

### Eligibility criteria

Inclusion criteria:

- Studies involving persons with limb amputation (upper or lower limb).
- Use of XR interventions (virtual reality, augmented reality, mixed reality, hybrid).
- Outcomes related to rehabilitation or user experience, including:
  Phantom limb pain.
  Functional task performance (e.g., walking, standing).
  Prosthesis control.
  Neurofunctional outcomes.
  User experience, engagement, or acceptability.
- Studies in human participants.
- Study designs including:
  Randomized controlled trials (RCTs).
  Non-randomized studies.
  Pilot studies.
  Case studies.

Exclusion criteria

- Studies not including XR-based serious games.
- Studies not involving people with limb amputation.
- Studies not including relevant rehabilitation and user-centred outcomes.
- System or technology development studies without human participants.
- Studies using synthetic data.
- Studies without reporting outcomes.
- Abstracts or reports without full manuscripts.
- Studies in languages other than English.

### Outcome domains aligned with research objectives

Outcomes were grouped *a priori* to allow synthesis of the diverse body of evidence. Our categories were defined to reflect the focus of XR-based rehabilitation interventions and variables extracted from studies. Five outcome categories were used: reduction in phantom limb pain, function, prosthesis control and motor performance, neurofunctional outcomes, and user experience and engagement.

*Phantom limb pain (PLP).* Phantom limb pain outcomes were defined as reductions in pain intensity or severity following XR-based interventions. These included studies that reported pain outcomes using standardized measures (e.g., numerical rating scale, questionnaire).

*Functional outcomes.* Functional outcomes included measures of physical performance and mobility, especially in lower-limb amputations. This included measures of gait, balance, walking, and mobility based on clinical tests.

*Prosthesis control and motor performance.* This domain included outcomes related to prosthesis use and motor performance, such as object manipulation, grasp performance, completion time, and motor coordination in XR.

*Neurofunctional outcomes.* Neurofunctional outcomes were defined as physiological or neurological changes following XR-based interventions, including indicators of cortical activity, embodiment, and other measures of sensorimotor adaptation.

*User experience and engagement.* User-specific outcomes included measures of usability, engagement, motivation, adherence, and workload. These were measured using validated scales such as the System Usability Scale (SUS) or Game Experience Questionnaire (GEQ), where reported.

*Outcome classification approach.* An included study could address 1 or more of the outcome categories based on the outcomes reported. This multi-dimensional classification approach enabled the mapping of XR rehabilitation impacts across clinical, functional, and user-centred domains, and accounted for the variety of study designs and outcome measures.

### Data extraction and synthesis

A data extraction table was developed to allow for meaningful comparisons. Studies were grouped by XR modality, game mechanic, control interface, and outcome domain. Quantitative pooling was not feasible given the heterogeneity of modalities, outcomes, and populations. Data were visualized using Microsoft Excel (Microsoft Corp, Redmond, WA, USA) and Flourish Studio (Flourish, London, UK). In the dot plot of percentage pain reduction, the midpoint was used as the plotted value where a range of scores was reported, with original bounds retained for interpretation and error bars where applicable. For 2 reported values, their midpoint was used as the representative estimate. No transformations were applied beyond these standardizations.

### Definition of serious games

Serious games were defined for the purposes of this review as interactive digital games primarily created for purposes other than entertainment, such as for rehabilitation, motor training, or therapy. For XR-based interventions in people with limb amputation, serious games involved immersive and non-immersive systems that included goal-directed tasks (e.g., object manipulation, balance, motor execution tasks, or simulations of activities of daily living), game mechanics (e.g., scoring, feedback, advancement, challenges), and interactive feedback controls (e.g., motion tracking, myoelectric [EMG] controls, or controllers) to support motor learning and rehabilitation. Both tailor-made rehabilitation games and commercial exergames (e.g., motion and/or sport simulation games) modified for rehabilitation purposes were included.

### Quality appraisal

The Mixed Methods Appraisal Tool (MMAT) (24) was used to evaluate the quality of studies included in this review. MMAT was chosen as it can be used to include heterogeneous study designs in the same appraisal framework and is therefore suitable for systematic reviews including quantitative, qualitative, and mixed-methods evidence. Each study was first assessed against 2 universal screening questions: (*i*) whether a clear research question was stated, and (*ii*) *whether the data collected could answer the research question*. Articles failing both screening questions were flagged but retained, in line with MMAT guidance that quality appraisal informs interpretation rather than serving as an exclusion criterion (24).

Each study was then classified by design and rated on the 5 design-specific MMAT criteria (*Yes*/*No*/*Unsure*), with a summary score out of 5. Two reviewers resolved discrepancies by consensus.

### Certainty of evidence (GRADE)

The certainty of evidence for each pre-specified outcome domain was assessed using GRADE (Grading of Recommendations Assessment, Development and Evaluation) methodology (25, 26). GRADE assessments were conducted *a priori* across 5 outcome domains: (*i*) PLP, (*ii*) functional outcome including balance, gait, and walking capacity, (*iii*) control and training performance of the prosthesis, (*iv*) usability and user experience, and (*v*) embodiment and neurofunctional outcome. Risk of bias drew on the MMAT appraisal. Inconsistency was assessed narratively given the heterogeneity of outcomes. Where there were substantial variations between the study populations, interventions, or outcome measures and the target population of adults with amputation receiving XR-based rehabilitation, *indirectness* was indicated. The rating of *imprecision* depended on sample size, availability of confidence intervals, and the width of effect estimates. Publication bias was considered likely across all domains, given the predominance of small, single-centre feasibility studies with consistently positive outcomes. The overall certainty of evidence for each domain was rated as high, moderate, low, or very low.

## RESULTS

### Study selection and overview

A total of 1,032 records were identified in the database search. After removal of duplicates and clearly ineligible records, 812 records were screened by title and abstract, of which 718 were excluded.

Ninety-four articles were retrieved for full-text assessment of eligibility. After full-text review, 70 studies were excluded (no XR/serious-game intervention, no amputees, no rehabilitation outcomes, simulation-only data, or no outcome reporting).

In total, 24 studies satisfied our inclusion criteria and were reviewed. The PRISMA flow diagram shows the study selection process (Fig. 2). A summary of the included studies is available in Table SII.

**Fig. 2:**
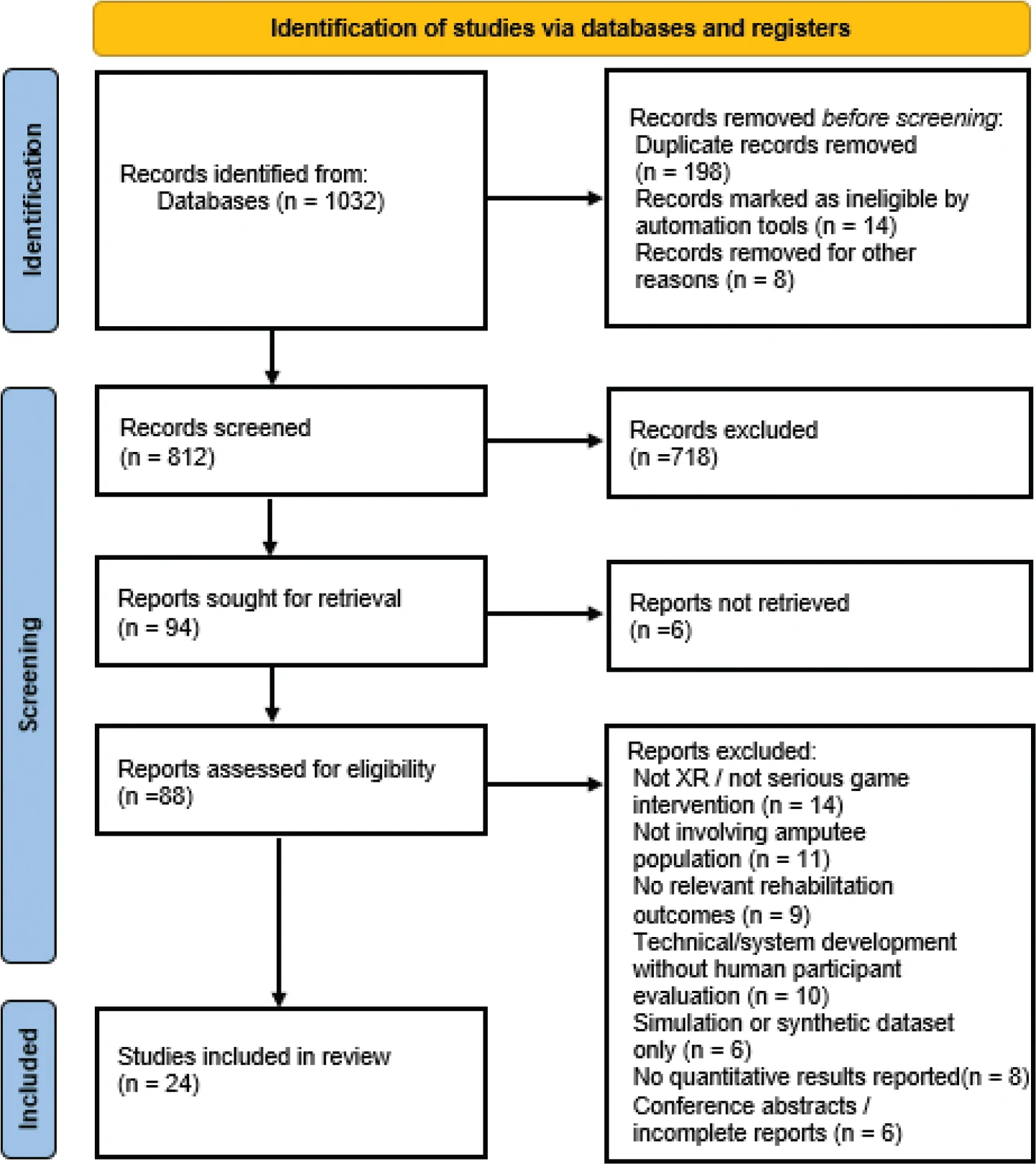
PRISMA 2020 flow diagram summarizing the study selection process, from database identification to final inclusion of eligible studies.

Most participants were individuals with amputation. Six studies (12, 27–31) enrolled a mix of healthy participants and participants with amputation, recruiting healthy participants for system testing or comparison; the remaining studies enrolled only participants with amputation.

Studies came predominantly from high-income countries, most often the USA (*k* = 6) (27, 32–36), followed by Canada (37–39) and Sweden (40–42) (*k* = 3 each). Thirteen studies were externally funded (28, 31, 32, 35–37, 39–45).

### XR game design and task category

XR rehabilitation interventions used a range of game-based approaches. Virtual reality (VR) was the most common and was used for prosthesis training, motor rehabilitation, phantom limb therapy, and Activities of Daily Living (ADL) tasks (27, 34, 44, 45).

Non-immersive VR was used mainly for balance and gait training in lower-limb amputees using motion-based exergames (37, 39, 43, 46, 47). Likewise, Pérez et al. (30) adopted a VR exergame-based system for rehabilitation where the patient completed lower-limb exercises by interacting with an avatar through robot-sensor-based body movements and immersive feedback based on the score.

Mixed and augmented reality was primarily used for phantom limb pain management and motor reorganization, typically involving EMG-based control of a virtual limb (12, 42, 48).

In general, the most frequent interaction modes were EMG-controlled interaction, body-based interaction (via body sensors), and controller-based interaction, which often used gamified feedback (scores, rewards, or other visual cues) to promote engagement and motor learning.

### XR technology, game mechanics, and rehabilitation outcomes

*XR modality by outcome domain.* The selection of XR modality was closely tied to the level of amputation and to the XR domain, and there were 2 distinct research traditions in the XR literature:

Immersive virtual reality (VR) through head-mounted displays (HMDs) such as HTC Vive, Oculus Rift, and Meta Quest was the most frequently used modality (*k* = 14) and was applied across the broadest range of tasks. For upper-limb studies, the application of immersive VR technology included prosthesis control training (27, 29) and phantom limb motor execution therapy (34–36), as well as object manipulation and grasping tasks (27, 31, 49). VR was used to aid motor rehabilitation, ADL training, and exercises using prosthetic legs in lower-limb studies (28, 30). In both populations, immersive VR was most strongly associated with prosthesis control and motor performance outcomes; it also reduced PLP through distraction and motor execution paradigms, which operate via different mechanisms.

Non-immersive VR exergames were applied only in lower-limb amputation studies for balance and gait rehabilitation and were mostly based on the Nintendo Wii Fit and Microsoft Kinect platforms (38, 39, 43, 46, 47, 50). The studies mostly focused on functional mobility outcomes such as gait speed, balance, and walking capacity, and formed the most methodologically robust subgroup in this review, including RCTs (39, 47).

Three upper-limb studies used AR or AR/VR hybrid systems delivering phantom motor execution (PME) (41, 42, 48), in which volitional myoelectric activation of residual-limb muscles controls a virtual limb to generate congruent sensorimotor feedback. This mechanic produced the largest effect sizes in the review (Cohen’s *d* = 0.9–1.3) (43, 49) and was the only one linked to neuroimaging evidence of partial cortical-reorganization reversal (49).

Three studies were conducted with both upper- and lower-limb populations, using mixed reality (MR) systems, such as the Microsoft HoloLens 2 (12, 32, 33). Both phantom limb therapy and exercises with the prosthesis were performed in ecologically valid environments using MR platforms, which uniquely integrated immersive virtual content with real-world spatial anchoring. The highest System Usability Scale (SUS) score in the review was reported for the PhantomAR system (90.8 [6.88]) (12), which also demonstrated peripheral physiological effects of embodiment (increased skin temperature at the residual limb) associated with sympathetic nervous system activation arising from virtual limb ownership. Values are reported as means (SD). MR systems therefore provided evidence in 3 areas: PLP reduction, embodiment and neurofunctional outcomes, and real-world adoption (usability).

*Game mechanics by outcome domain.* Five main categories of game mechanics were found: (*i*) object manipulation and grasping games; (*ii*) balance and postural control games; (*iii*) target-directed motor execution games; (*iv*) ADL simulation games; and (*v*) sports and movement exergames. The mapping between mechanic type and outcome domain was consistent and theoretically coherent.

Studies on upper-limb prostheses training focused on object manipulation and grasping tasks, such as Block Builder, Cup Transfer, and pick-and-place paradigms, primarily yielding prosthesis control and motor performance outcomes such as a decrease in task completion time, increased accuracy on the grasp selection task, and improvements in Box and Block Test scores (27, 29, 45). In these tasks, EMG biofeedback was the primary control interface, and outcome specificity was high.

Balance and postural control games, such as Wii Fit balance exercises, Kinect obstacle-crossing, and VR sports simulations, were employed to assess lower-limb outcomes and were the only mechanics in this review that yielded functional mobility outcomes. Several studies noted improvements in Berg Balance Scale (BBS), Dynamic Gait Index (DGI), 6-Minute Walk Test (6MWT), and Timed Up and Go (TUG) with 2 RCTs (39, 47) supporting these findings most strongly.

A few studies focused on the upper limb, using ADL simulations (e.g., virtual kitchen task, coffee-making simulation), which contributed primarily to prosthesis control outcomes through their ecological validity, and secondarily to user experience outcomes (31, 43, 47). For lower-limb studies, sports and movement exergames (e.g., Kinect football, tennis simulations) had a positive impact on functional mobility, and were linked to positive engagement and adherence outcomes (43, 47).

*Gamification design and outcome relevance.* Score-based feedback was the most common mechanic (reported in 13 studies) and was present in all 5 outcome domains, across all categories of mechanic. This ubiquity, however, reflects a design constraint rather than deliberate optimization: score feedback was often a default feature of commercial or modified game platforms rather than an intentional design choice.

The exergame-based studies in the lower limb (37–39, 43, 46, 47) employed mechanisms of progressive difficulty (51), consistent with motor learning theory and the challenge-point framework, in which optimal task difficulty for both acquisition and retention occurs when difficulty is matched to the individual’s current ability. However, adaptive difficulty based on within-session performance data was rarely used; in most studies, difficulty was preset or therapist-controlled.

Reward-based gamification (e.g., virtual coins, level-ups) was applied in only 2 studies, both related to prosthesis training (27, 31). In the PhantomAR system (12), open exploratory interaction with virtual objects was used for phantom limb therapy; this approach was not explicitly compared with structured approaches but is theoretically linked to intrinsic motivation.

Table I provides a summary of the relationships between the modality of XR, the type of game mechanics, the primary mode of control, and the outcome domain in which evidence could be identified in the 24 included studies.

**Table I:** XR modality, game mechanic, control interface, and primary outcome domain across included studies

XR modality	Primary game mechanic	Control interface	Primary outcome domain	Representative studies
Immersive VR (HMD)	Object manipulation; ADL simulation	EMG; VR controller	Prosthesis control; PLP reduction	(27, 31, 34, 36)
Non-immersive VR exergame	Balance games; sports simulation	Balance board; motion tracking (Kinect)	Functional mobility	(37–39, 43, 46, 47)
Augmented Reality (AR)	Target-directed motor execution	EMG myoelectric	PLP reduction; neurofunctional reorganization	(41, 42, 48)
Mixed Reality (MR)	Free phantom limb interaction; lower-limb exercise	EMG; IMU sensors; motion tracking	PLP reduction; embodiment; usability	(12, 32, 33)

HMD: head-mounted display; EMG: electromyography; IMU: inertial measurement unit; ADL: activities of daily living; PLP: phantom limb pain.

### Game design feature analysis

*Gamification elements.* Cross-analysis of gamification elements and outcomes showed that score-based feedback was the most common design feature across outcome categories, especially in studies focused on pain relief and mobility improvements (Fig. 3A). For instance, score or performance feedback was frequently used in VR-based rehabilitation tasks and games for phantom limb therapy (28, 34, 41, 43, 48).

**Fig. 3:**
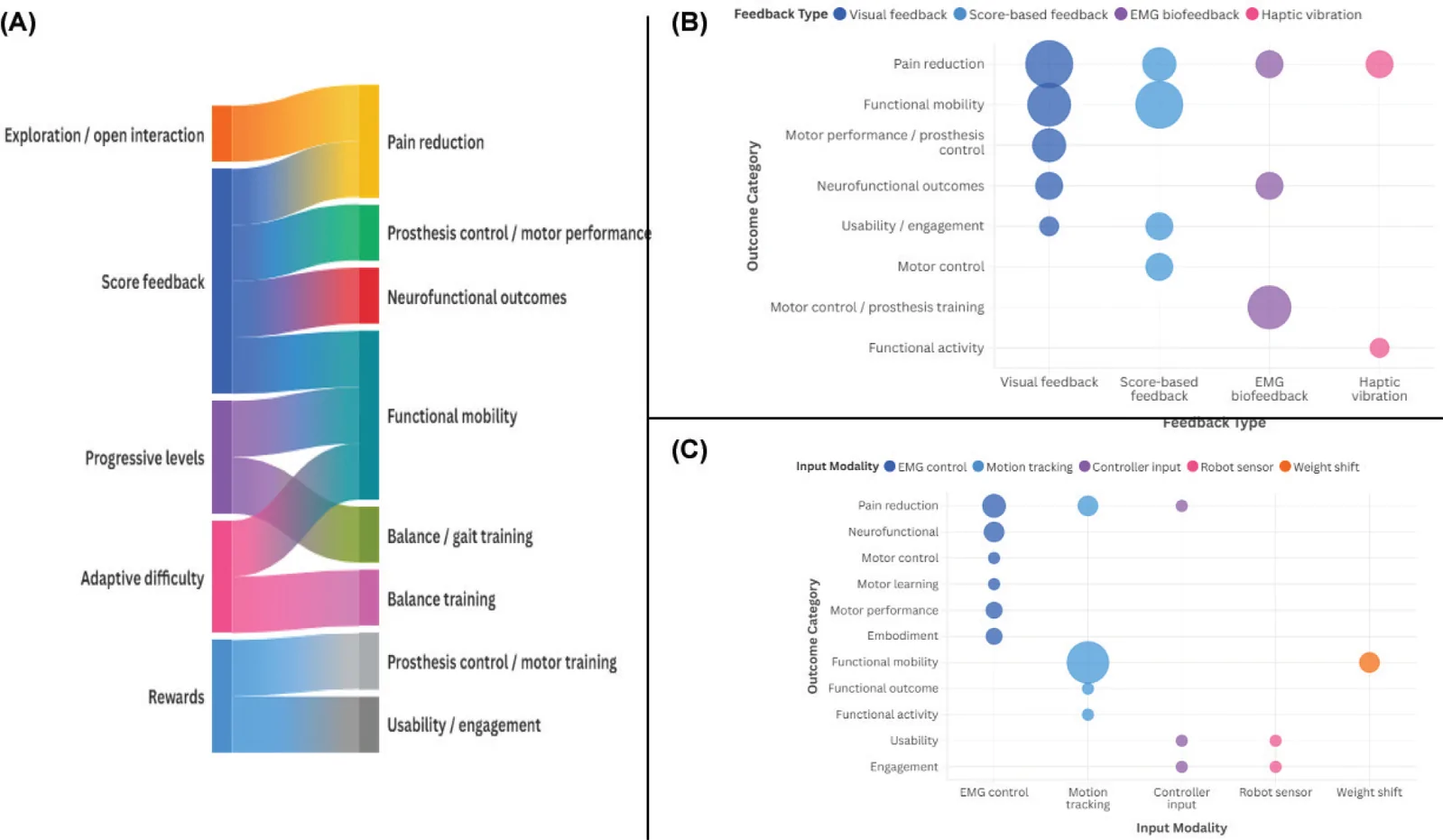
(A) Sankey diagram showing cross-analysis of gamification elements and outcomes. (B) Relationship between feedback modalities and rehabilitation outcomes in serious games for amputation rehabilitation. (C) Evidence map of interaction modalities and rehabilitation outcomes in serious games for amputation rehabilitation.

Progressive levels and adaptive difficulty were primarily found in exergame-based balance and gait training, using Wii Fit or sports games (37–39, 43). By contrast, reward-based gamification elements were seldom used, and mainly in the context of prosthesis training systems or virtual-reality video games for rehabilitation (31, 45).

*Feedback type analysis.* Visual feedback was the most prevalent modality across all rehabilitation outcome domains, reflecting its broad applicability regardless of amputation level or XR platform. EMG biofeedback, by contrast, was concentrated in prosthesis training and phantom motor execution systems, where real-time representation of myoelectric signals was central to the therapeutic mechanism. Haptic vibration feedback was rarely implemented across the corpus, appearing in only a small number of studies, and no study combined all 3 feedback modalities within a single intervention. Bubble sizes in Fig. 3B reflect the number of studies reporting each feedback–outcome combination, illustrating that the evidence base for non-visual feedback remains thin relative to its theoretical relevance for embodiment and sensorimotor rehabilitation.

*Control interface analysis.* EMG-based control and motion tracking were the most common interaction modalities, reported in 9 and 8 studies respectively, and served functionally distinct rehabilitative roles. EMG interfaces were coupled primarily with upper-limb prosthesis training and phantom motor execution tasks requiring volitional myoelectric activation (12, 27, 29, 31, 36, 41, 42, 45, 48), while motion tracking underpinned VR and exergame systems targeting functional mobility via Kinect or inertial sensors (32–35, 43, 44, 46, 47). A single study used robotic position sensors embedded in a rehabilitation device (30) (Fig. 3C). Controller-based interaction was confined to a subset of immersive VR environments (45, 49), and weight-shift input via balance boards appeared exclusively in Wii Fit-based walking rehabilitation programmes (37–39). Across modalities, the pairing of input interface with outcome domain was consistent: myoelectric control was associated with upper-limb prosthesis and pain outcomes, while motion-based and weight-shift interfaces were associated with lower-limb functional mobility outcomes.

### Dose–response analysis of game-based training

The dose–response parameters varied among the included studies, but some general trends emerged. Session duration ranged from about 7–90 min, with the most common programme using 30–60 min per session (12, 27, 31, 32, 37–40, 43–49). Most studies included 3 sessions per week, but session frequencies varied from 1 per month to 5 per week (31, 32, 37–40, 43, 46–48). The duration of the intervention ranged from 2–52 weeks, with 4 weeks being the most frequently reported length of programme. Using the described parameters, the cumulative intended rehabilitation dose ranged from about 60–960 min (12, 27, 31, 37–39, 43–48). Lower-limb rehabilitation programmes typically involved longer durations and higher doses, as repeated practice is required for training walking and balance skills (37–39, 43, 46, 47), while upper-limb interventions (i.e., those targeting the control of a prosthesis, or phantom limb pain) typically involved shorter durations and lower doses (12, 27, 31, 44, 45, 48, 49).

To calculate the total dose (reported in minutes) for studies that did not explicitly report a cumulative intervention value (31, 37–39, 43, 44, 46, 48), we used the following equation:

*Total Dose* (*min*) = *Session Length* (*min*) × *Sessions per Week* × *Program Duration* (*weeks*)

This consistency allows us to compare the dose of different XR rehabilitation programmes, whether the original study reported daily session length or duration of the programme.

The bubble plot (Fig. 4A) shows intervention duration and session length across studies. Bubble size represents the intended total dose of the intervention and colours represent different types of amputation studies (upper and lower limb). Lower-limb intervention programmes tended to have longer durations and longer total exposure to training compared with upper-limb programmes.

**Fig. 4:**
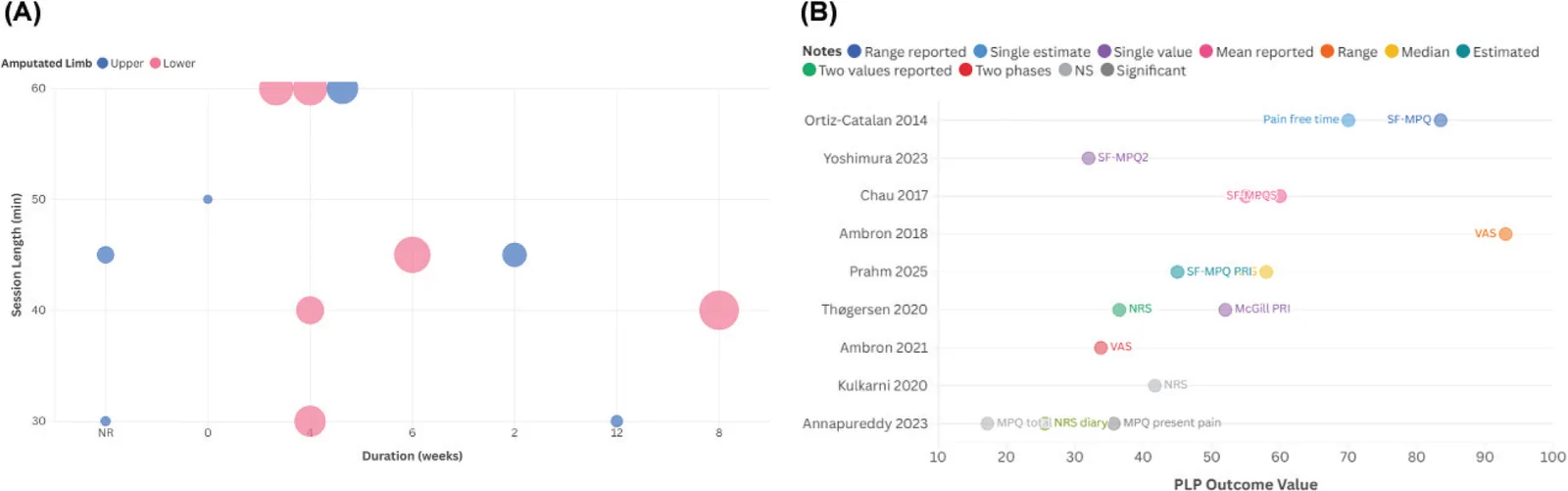
(A) Dose–response landscape of XR rehabilitation interventions for amputees. (B) Dot plot of percentage reduction in phantom limb pain (PLP) across included studies. Each point represents a study–outcome pair, with midpoint values plotted for ranges and original values retained for single estimates. Colours denote reporting type (e.g., mean, median, range), illustrating variability in effect magnitude and outcome measurement across XR-based interventions.

This dose–response pattern shows that higher-dose and longer-duration XR programmes were primarily associated with functional rehabilitation outcomes, particularly gait and balance training in lower-limb amputees, whereas lower-dose and shorter-duration interventions were more commonly used for PLP, prosthesis control and motor performance, neurofunctional adaptation, and user engagement outcomes in upper-limb rehabilitation contexts.

### Phantom limb pain analysis

Ten studies reported quantitative pain outcome data in individuals with phantom limb pain (PLP) treated using XR-based interventions, encompassing a range of instruments including the Numerical Rating Scale (NRS), Visual Analogue Scale (VAS), Short-Form McGill Pain Questionnaire (SF-MPQ and SF-MPQ-2), the full McGill Pain Questionnaire (MPQ), and the Weighted Pain Distribution scale (WPD). Substantial heterogeneity in scale type, measurement timepoints, intervention design, session frequency, and sample characteristics precluded meta-analytic pooling; findings are accordingly reported narratively by design category.

Within-session analgesic effects were consistently substantial across immersive VR studies, with reductions of 55–60% on VAS and SF-MPQ scores reported during sessions, including a 90% reduction on the Wong–Baker FACES scale (*p* = 0.0024) with pain relief lasting approximately 24 h post-session (36), and near-complete pain elimination observed in small case series (pre-session pain reductions of 22% and 67% across sessions in 2 lower-limb cases, with 5 of 6 post-session pain scores reaching 0/10 [35]), though post-session maintenance was inconsistently examined. In the study by Ortiz-Catalan et al. (42) (*n* = 14, 12 AR-PME sessions), PLP decreased by 47% on the WPD (*p* = 0.001), 32% on the NRS (*p* = 0.007), and 51% on the PRI (*p* = 0.0001). Intrusion into ADL dropped 43% (*p* = 0.004) and into sleep 61% (*p* = 0.001). Two of 4 medicated patients reduced their medication intake by 81% and 33%, respectively. “Stabbing” and “tiring-exhausting” qualities became significantly less prevalent (*p* = 0.016). Improvements in WPD and NRS were maintained at 1-, 3-, and 6-month follow-up. Yoshimura et al. (49) (single-case ABA’B’ design, 42-year-old with 9-year post-amputation PLP) reported SF-MPQ-2 scores of 147 → 128 → 93 → 100/220 across Periods A, B, A’, and B’. Gnawing, piercing, punishing-cruel, and tiring-exhausting qualities resolv-ed completely (8 → 0), while throbbing, stabbing, and sharp pain returned in Period B’. Across both time horizons, effect magnitudes were larger for affective and intensity dimensions of pain than for somatosensory qualities, a pattern consistent with the proposed mechanism of sensorimotor cortical reorganization underlying AR-based PME approaches. Annapureddy et al. (Mr. MAPP, *n* = 11, 1-month home MR) reported MPQ current-pain reduction from 1.75 to 1.125/5 (*p* = 0.011), with significant drops in the constrictive (3.75 → 2.0, *p* = 0.049) and evaluative (2.125 → 1.625, *p* = 0.033) subclasses. Weekly pre-exercise pain fell 25% by week 4 (*p* = 0.019) (32). Methodologically weaker evidence of benefit was provided by uncontrolled group studies. Ortiz-Catalan et al. (42) provided the strongest results. At follow-up, PRI was 2%, 6%, and 24% below the last-session value at 1, 3, and 6 months. Similar findings were noted in smaller groups, such as decreases in PRI and NRS of approximately 52% and 41%, respectively, and neuroimaging evidence of decreased maladaptive cortical activity (12, 48). Single-session or short-term interventions also showed significant decreases (e.g., a 58% NRS decrease [12]). Other reports showed medium though inconsistent improvements (e.g., 28–40% VAS or 36% MPQ reduction in Ambron et al. [34] and Annapureddy et al. [32] respectively), or non-significant or unstable improvements (44). Longitudinal home-based data also highlighted large inter-individual differences: across 4 amputees followed over 12 months, monthly adherence ranged from 0% to 137.5% of the recommended 8 sessions, with 1 participant phasing out by month 7 and another sustaining 25–87% throughout, while session durations mostly fell below the recommended 90 min (40). Yoshimura et al. additionally quantified real-world limb use via 24-h accelerometry: amputated-limb activity rose from 1,076,658 vector-magnitude units in Period A to 2,542,317 in Period A’, reaching parity with the intact limb and enabling resumption of bimanual ADLs (drying clothes, cooking, writing, carrying a grandchild) (49). Lip-pursing functional magnetic resonance imaging (fMRI) (48) showed a significant decrease in S1 activity contralateral to the amputation (Brodmann area [BA] 3b; *p* = 0.011), with the cortical lip representation shifting 4.62 mm (SD = 3.87) toward the healthy-side location (*p* = 0.056) after 2 weeks of AR training. None of the 10 PLP studies included a no-intervention or active comparator arm. All effect sizes are within-subject changes from baseline.

### Functional mobility outcomes

Two RCTs provided the most methodologically robust functional-mobility evidence in the corpus. Abbas et al. (43) (*n* = 32, 6-week VR + exercise vs exercise) found significant between-group gains favouring VR on DGI (17.81 → 22.75; *p* = 0.0001), TUG (11.11 → 8.05 s; *p* = 0.001), and BBS (43.19 → 51.38; *p* = 0.035); the 6MWT improved within-group (166.19 → 262.63 m) but not between groups (*p* = 0.091). Imam et al. (39) (Wii.n.Walk, *n* = 28) reported adherence of 83.4%, perceived benefit 38.9/45, 2MWT Cohen’s *d* = 0.5–0.6 (intention-to-treat) and 0.9–1.2 (per protocol), and Short Physical Performance Battery (SPPB) *d* = 0.7–0.8. Şahan and Erbahçeci’s RCT (47) (*n* = 20 transtibial amputees; 4-week Kinect Xbox VR vs physiotherapy) likewise showed significant within-group gains in both arms across performance, balance, prosthesis satisfaction, cadence, and gait speed (all *p* < 0.05) but no overall between-group difference (*p *> 0.05); median 6MWT distance rose from 227.65 to 263.92 m (VR) and from 228.60 to 387.90 m (PT), with between-group differences emerging only for prosthetic-limb single-leg balance at weeks 3–4 (*p* = 0.029, 0.028). Complementing these trials, Tao et al.’s WiiNWalk analysis (37) (*n* = 36 experimental, *n* = 28 control) found unsupervised/supervised session-count ratios below parity in both groups (0.25 vs 0.18; between-group *p = *0.92) but session-time ratios favouring the experimental arm (1.12 vs 0.76; *p* = 0.027). Smaller feasibility studies reinforced the pattern: Imam et al.’s WiiFit SSRD (38) (*n* = 6) reported 80% adherence and statistically significant 2MWT improvement in 5 of 6 participants (final median 166 m), with SPPB gains in 4/6 and ABC in 3/6; Annaswamy et al.’s Mr. MAPP pilot (33) (*n* = 4 completers; 57% retention) showed PSFS improvement in 2/3 participants (overall 4.625 → 5.75) but no clear pain-score trend; and Pérez et al.’s Kina/Exogames evaluation (30) (*n* = 6) reported a SUS score of 69 (66, 79), PACES 110/126 (108, 112), and GEQ immersion 4 (3, 5), supporting usability and engagement. Collectively, VR interventions produced consistent within-group functional gains and favourable adherence, with between-group superiority over conventional therapy demonstrated most clearly in Abbas et al. (43) and on selected balance measures elsewhere.

### User experience and engagement analysis

Usability ratings, measured using the System Usability Scale (SUS), were consistently high across upper-limb XR rehabilitation systems. Three upper-limb studies reported mean SUS scores of 81.9 (10.0) (27), 90.8 (6.88) (12), and a score above 70 (45), reflecting acceptable to excellent perceived usability among both prosthesis users and therapists. The single lower-limb study reporting SUS showed a notably lower median score of 69/100 (30), suggesting a potential usability gap between upper- and lower-limb rehabilitation platforms. Therapist-reported workload, assessed via the Prosthesis Task Load Index (PROS-TLX) in one study (45), was higher than that of prosthesis users (38.3 vs. 23.0), with significant differences in visual attention (95.0 vs 50.0, *p* = 0.04) and conscious processing (91.0 vs 60.0, *p* = 0.008) subscales, though overall workload did not differ significantly between groups (U = 83, *p* = 0.15).

Engagement and motivational outcomes were positive across studies. GEQ scores in 1 upper-limb study (12) indicated high immersion (4.46 [0.08]) and positive affect (4.97 [0.05]), and a lower-limb exergame study (30) reported similarly favourable profiles across flow, positive affect, immersion, and competence (all rated 4/5), with low tension and negative affect (both 1/5). Intrinsic Motivation Inventory (IMI) scores from 1 prosthesis training study (31) showed high interest and enjoyment (6.6 [0.49]), perceived competence (6.2 [0.40]), and value/usefulness (6.2 [0.40]), with moderate perceived choice (4.4 [0.49]) and low pressure/tension (2.0 [0.63]). Hashim et al. (31) also reported a strong positive correlation between training period and Box and Block Test score (*r* = 0.95, *p* < 0.001). A single-case VRT study (49) reported VAS immersion of 95.2 (9.6) (Period B) and 100 (0.0) (Period B’) and sense-of-agency Likert scores of 6.4 (0.8) and 7.0 (0.0). Together, these findings indicate that XR interventions were well tolerated and intrinsically motivating across amputation levels and modalities.

Adherence was reported in 2 lower-limb exergame studies, with rates of 83.4% and 80%, respectively (38, 39), suggesting acceptable engagement with home- or clinic-based programmes. Dropout and attrition were reported across several studies, with causes including logistical difficulties, health problems, prosthesis discomfort, and equipment availability (33, 34, 38–40, 43, 44). Adverse events were rare and mild, limited to transient nausea during immersive XR exposure in 2 studies (38, 48).

### Analysis of XR rehabilitation systems by amputation level

Among upper-limb studies (*k* = 11), 9 of 11 used myoelectric (EMG) control, 1 used controller-based interaction, and 1 used intact-limb motion tracking (12, 27, 29, 31, 36, 41, 42, 45, 48).

A Sankey diagram was developed to investigate the association between amputation level, XR technology, modality of interaction, and clinical outcomes (Fig. 5). The diagram depicts the evidence flow through 4 layers, including amputation level, type of XR technology, input modality, and outcome category. The most frequently used upper-limb rehabilitation systems were augmented or virtual reality environments with EMG-based control interfaces, targeting reduction of phantom limb pain, embodiment, and neurofunc-tional outcomes. Conversely, VR exergames with motion-tracking or weight-shifting input were the main interventions in lower-limb rehabilitation, targeting functional mobility, gait, and balance.

**Fig. 5:**
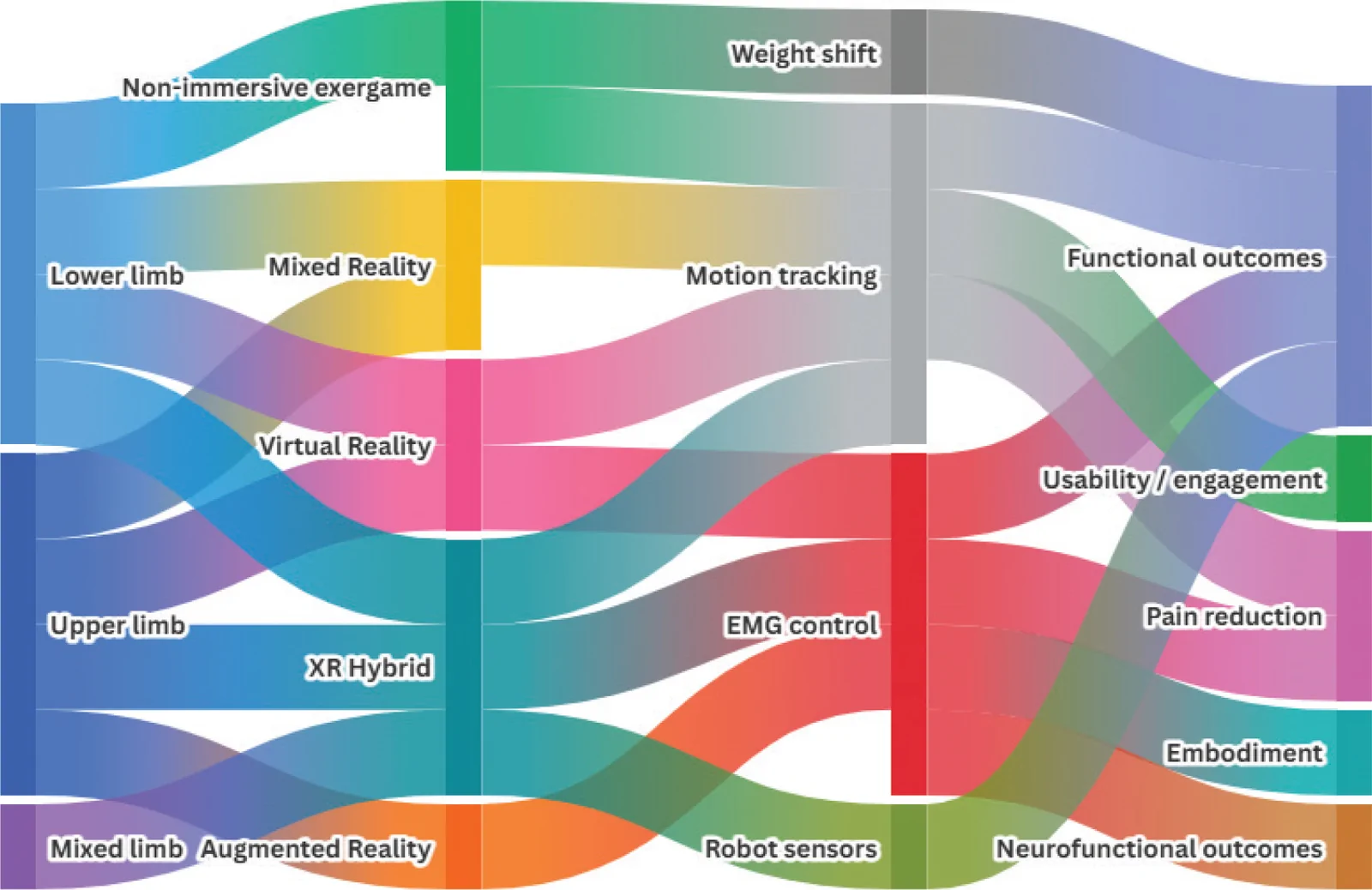
Sankey diagram illustrating relationships between amputation level, XR modality, interaction interface, and outcome domains.

### Risk of bias

All 24 included studies were appraised with the MMAT against 2 universal screening questions and 5 design-specific criteria, each rated Yes, No, or Unsure. Studies fell into 4 design categories: randomized controlled trial (RCT; k = 2), quantitative non-randomized (*k* = 5), quantitative descriptive (*k* = 13), and qualitative (*k* = 2, including case reports). Table SIII presents summary scores out of 5.

The general methodological quality of the included studies, assessed with the Mixed Methods Appraisal Tool (MMAT), was low to medium. Scores ranged from 0/5 to 5/5, with most of the studies (*k* = 11) scoring 1–2/5. Only 3 studies achieved scores of 4/5 or above (39, 42, 43). Imam et al. (39) scored 5/5 as the sole RCT with confirmed block randomization, allocation concealment, evaluator blinding, and intention-to-treat analysis. Abbas et al. (43) scored 4/5 with comparable rigour but without explicit confirmation of allocation concealment. Ortiz-Catalan et al. (42) scored 4/5 in the non-randomized category, distinguished by a multicentre design, validated multidimensional pain outcomes, and 6-month follow-up. Annaswamy et al. (33) had a 43% dropout rate and reported neither effect estimates nor an analysis of missing data. A systematic methodological constraint across the corpus was the use of non-representative mixed samples that included able-bodied participants (12, 27, 29, 31).

### Certainty of evidence (GRADE)

Evidence was evaluated across 5 domains: risk of bias, inconsistency, indirectness, imprecision, and publication bias. Because most of the included studies were uncontrolled observational or descriptive studies, evidence on all outcomes started at low or very low certainty (Table SIV).

The highest certainty rating in the review was for functional outcomes, such as balance, gait, and walking capacity (low), which were supported by 2 RCTs (39, 43) with assessor blinding and intention-to-treat analysis. Directional consistency across studies prevented further downgrading for inconsistency. Usability and user experience were also rated as low certainty, although SUS scores were consistently above the accepted threshold of 70 in 3 independent samples, all were self-reported, and no active comparators were used.

The remaining outcome domains – PLP reduction, prosthesis control and training performance, and embodiment and neurofunctional outcomes – were all rated very low. PLP results were downgraded in all 5 GRADE domains: no RCTs contributed evidence, samples were small and heterogeneous, measurement instruments varied, and confidence intervals were not reported. Prosthesis training results were further downgraded for indirectness, as most contributing studies recruited predominantly able-bodied participants rather than individuals with amputation. Embodiment outcomes were limited to single studies using non-standardzed instruments without control conditions, precluding pooling of evidence.

## DISCUSSION

### Principal findings

This review synthesised 24 studies (2013–2026) across 5 outcome domains. XR-based serious games appear acceptable to users and capable of reducing PLP and improving function in both upper- and lower-limb amputees.

Clear patterns of evidence emerged in each of the 5 pre-specified outcome domains, with findings across domains showing convergent directional consistency. Findings for XR interventions in PLP showed a consistent trend towards pain reduction, although evidence certainty was very low owing to methodological limitations and heterogeneity. Functional mobility outcomes had the strongest evidence base, with low-certainty evidence from 2 RCTs that VR exergame interventions led to improvements in balance, gait, and walking performance in lower-limb amputees. While the evidence was limited due to indirectness and sample size, the results indicated that immersive VR and EMG training systems could enhance object manipulation, task completion, and motor coordination, as well as prosthetic control. Neurofunctional and embodiment outcomes provided preliminary mechanistic evidence for potential effects of XR interventions on cortical reorganization, embodiment, and physiological adaptation, but certainty was very low due to a limited number of exploratory studies. User experience and engagement outcomes showed generally positive usability, immersion, and adherence across XR systems, with limited data on implementation and therapist workloads.

Nevertheless, the evidence certainty in all outcome domains is very low to low according to GRADE, hence these results should be taken with great caution. The evidence divides structurally by amputation level, a pattern not previously synthesized in the literature. Upper-limb rehabilitation studies predominantly employed AR- and VR-based systems with EMG-based myoelectric control, focusing on PLP, phantom motor performance, embodiment, and neurofunctional reorganization. Lower-limb systems centred on VR exergames and motion-tracking interfaces, focusing on functional mobility, gait, and balance. This bifurcation has direct implications for the design of future trials, the selection of outcome measures, and the prioritization of technology development resources.

### Technological evolution and the serious game design framework

The 4-stage development pattern identified in this review – from commercial exergames (Phase 1: 2013) through early AR/VR therapy (Phase 2: 2014–2018) and sensing-integrated exergames (Phase 3: 2020–2021) to advanced XR prosthesis training (Phase 4: 2022–2026) – is consistent with broader trends in digital rehabilitation technology development (Fig. 6).

**Fig. 6:**
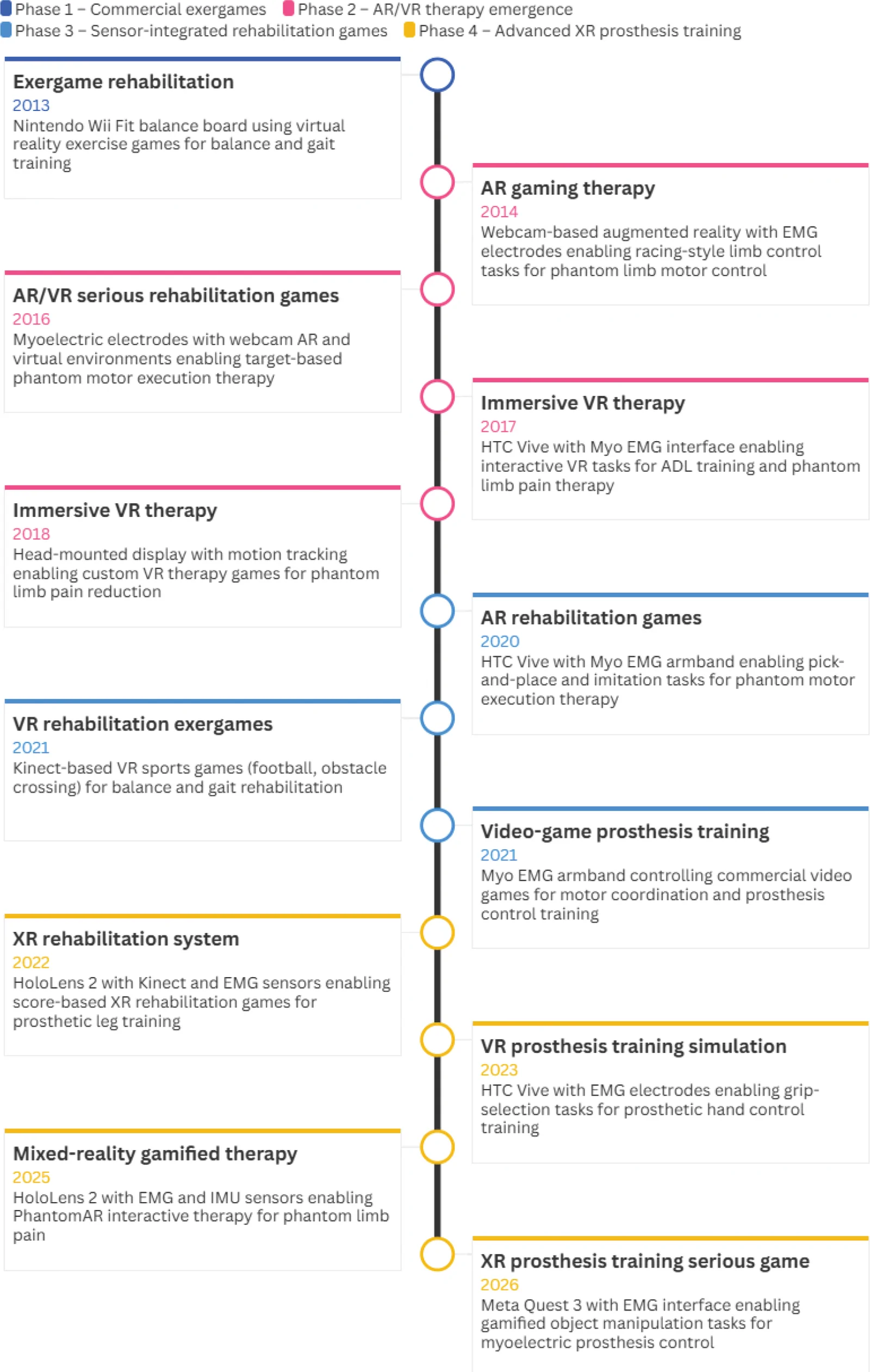
Evolution of serious games for amputation rehabilitation.

Notably, the current review shows that most existing systems are optimized at the platform and control-interface levels, whereas the game design layer is a relatively underdeveloped part of the system. The gamification aspects are mostly restricted to score-based feedback, whereas reward systems, adaptive difficulty, and narrative progression are seldom provided. Evidence from motor rehabilitation more generally indicates that engagement-sustaining game features, such as challenge–skill balance, mastery progression, and social features, are independently linked to adherence and functional outcomes (52, 53).

### Phantom limb pain and XR serious games

All 10 studies reporting PLP showed a directional reduction in pain. As summarized in Results, AR-based phantom motor execution produced the largest and most durable effects (sustained to 6 months), while immersive VR single-session studies showed large within-session reductions whose durability was not consistently examined. Taken together, the pattern suggests that mechanism (not immersion alone) governs how long analgesic effects last.

These results are consistent with the maladaptive cortical reorganization hypothesis (54), according to which motor execution-based therapies that restore coherent sensorimotor feedback can partially reverse the pathological cortical reorganization. The neuroimaging sub-study by Thøgersen et al. (48) provided direct mechanistic support, as the observed reduction in contralateral S1 activity and the measured shift of cortical lip representation towards the healthy-side location are consistent with partial reversal of maladaptive cortical reorganization after only 2 weeks of AR-based training.

AR-based phantom motor execution uses volitional myoelectric control and closed-loop sensorimotor feedback, thereby engaging neuroplastic mechanisms, whereas VR distraction operates mainly through attentional diversion. The emergence of home-based platforms adds dosing flexibility, but high inter-individual variability underscores the need to identify clinical predictors of response.

### Neurofunctional and embodiment outcomes

Neurofunctional and embodiment outcomes were reported only in upper-limb amputation studies, reflecting the theoretical priority of motor representation and cortical adaptation in upper-limb rehabilitation. As summarized in Results, the strongest mechanistic signal came from AR-based phantom motor execution, where reductions in PLP covaried with increases in embodiment and agency and with felt telescoping, supporting the interpretation that volitionally coherent sensorimotor feedback (not distraction) drives the observed cortical and perceptual changes. Complementary mixed-reality evidence (increased ownership and reduced cortical activity) and peripheral physiological evidence (residual-limb skin-temperature changes with PhantomAR) extend this picture to autonomic and embodied-perception levels.

Prosthesis ownership has been linked to greater prosthesis use and lower rejection rates (55); XR-based embodiment protocols may therefore support improved prosthesis uptake.

### Functional mobility and prosthesis control outcomes

Functional-mobility outcomes received the strongest support in this review, with 2 RCTs (39, 43) showing significant gains on validated balance and gait measures (BBS, DGI, 6MWT, TUG) after VR-exergame training in lower-limb amputees. Upper-limb prosthesis-control gains in task-completion time, grip selection, and Box-and-Block scores were consistent, although their interpretability was limited by the use of able-bodied samples in several studies.

### Real-life adoption: usability, interaction, and adaptation

A notable finding of this review is the relative scarcity of user-experience and adoption data. Only 4 studies formally measured usability using the System Usability Scale, and only 2 studies reported adherence rates. This represents a notable gap: the field has prioritized clinical outcome measurement while implementation science outcomes remain underreported.

The available usability data are generally positive. Three out of 4 studies found SUS scores exceeding the 70-point level of acceptable usability, and engagement measures also reported positive immersion, competence, and enjoyment with low tension and negative affect. The disconnect between laboratory usability and real-world adoption, however, is, however, well recognized in rehabilitation technology (56). The reported causes of participant withdrawal included equipment supply issues, discomfort with the prosthesis, health problems, and logistical barriers. The elevated therapist workload on PROS-TLX visual-attention and conscious-processing subscales (45) points to an under-recognized scalability constraint, especially in resource-limited settings. Future systems should prioritize automation, intuitive calibration, and remote monitoring to ease clinician burden.

Implementation science frameworks can offer a structured approach to the translational challenge between promising XR prototypes and sustained clinical uptake. For instance, the Consolidated Framework for Implementation Research (CFIR) (57) might be used to determine barriers and facilitators related to the characteristics of the intervention, clinical settings, therapist perceptions, and organizational readiness, and the RE-AIM framework (Reach, Effectiveness, Adoption, Implementation, Maintenance) to evaluate long-term deployment outside of the laboratory setting (58). The use of these frameworks would enable future studies to go beyond usability reporting and assess the clinical performance of XR systems across a range of factors, such as cost, scalability, training needs, sustainability, and accessibility.

### Design recommendations for gamification and XR rehabilitation systems

Score-based feedback predominated across all outcome categories, whereas reward systems, adaptive difficulty, narrative progression, and social features were seldom applied. Adaptive difficulty is of particular interest: both optimal challenge theory and the contextual interference effect suggest that adjusting task difficulty to patient performance facilitates skill acquisition and retention.

Only 1 study (12) used haptic feedback, despite strong theoretical and empirical support for multisensory feedback in prosthesis embodiment and phantom-limb modulation (59). Integrating haptic interfaces into XR serious games is therefore a high development priority.

Collectively, these findings suggest that future XR rehabilitation systems should move beyond minimal score-based gamification towards theoretically informed designs incorporating adaptive difficulty (60), multisensory feedback, progression systems, and intrinsically motivating mechanics that are explicitly aligned with motor learning and neurorehabilitation principles.

### Limitations of the included studies

The overall methodological quality of the included literature was moderate to low, with most studies scoring 1–2 out of 5 on the MMAT. Pervasive methodological limitations included small and non-representative samples, absence of control conditions in most studies, heterogeneous outcome measurement, lack of blinding, and limited follow-up. The concentration of funded research in high-income countries, together with predominantly positive directional results, is consistent with publication bias. The adoption of pre-registered trial protocols, standardized outcome sets, and transparent reporting based on CONSORT (61) or similar guidelines would benefit the field.

### Limitations of the present review

There are also a few limitations to the present review. First, heterogeneity of modalities, protocols, outcomes, follow-up, and populations precluded meta-analysis. Second, the broad inclusion criteria that enabled landscape mapping limited direct between-study comparison. Third, publication bias is likely, given the predominance of small feasibility and pilot studies with generally positive results. Fourth, only studies in English were considered, which may have excluded relevant studies published in other languages. Finally, although this review proposed a conceptual model that connects neurofunctional mechanisms to embodiment and implementation outcomes, the current evidence base is insufficient to test all proposed pathways empirically.

### Future research and clinical practice implications

Adequately powered RCTs are needed for all outcome domains. Specifically, PLP trials should involve chronic PLP populations with pre-specified entry criteria, multidimensional pain measurements with known psychometric properties, standardized neuroimaging endpoints and cortical-reorganization indices to allow comparative meta-analysis, and at least 6 months of follow-up.

Dose-finding research is also required to establish optimal session duration, frequency, and programme length. Implementation science frameworks should be used to characterize barriers and facilitators to XR implementation in clinical environments, in both home-based care and low-resource settings.

### Conclusion

XR serious games show preliminary evidence of benefit for PLP, functional rehabilitation, prosthesis control, neurofunctional recovery, embodiment, and user experience after amputation. AR- and MR-based phantom motor execution produced the most consistent PLP reduction with mechanistically plausible neurofunctional support, while VR exergames produced the strongest functional-mobility gains, backed by 2 RCTs. Overall GRADE certainty remains low to very low across domains, and therapist workload and equipment logistics emerged as practical barriers to adoption. Future work should prioritize adequately powered RCTs, standardized neurofunctional and implementation outcomes, and neuroscience-informed game design.

## Supplementary Material


