# Supplementary material for: XR SERIOUS GAMES FOR PHANTOM LIMB PAIN, NEUROFUNCTIONAL RECOVERY, AND PROSTHETIC ADOPTION: A SYSTEMATIC REVIEW
Source: J Rehabil Med. 2026 Nov 13;58:46784. doi: 10.2340/jrm.v58.46784 (PMC13761211; doi:10.2340/jrm.v58.46784)
Supplement: Supplementary file 1 [file JRM-58-46784-s1.pdf]

Table SI. Boolean search strings.

Database	Platform	Search String
PubMed	MEDLINE	(("Amputation"[Mesh] OR amputat* OR "limb loss" OR "upper limb amputation" OR "lower limb amputation") AND ("Virtual Reality"[Mesh] OR "Augmented Reality"[Mesh] OR "Mixed Reality" OR "Extended Reality" OR VR OR AR OR MR OR XR) AND ("Video Games"[Mesh] OR "Serious Games" OR exergam* OR "game-based" OR "gamified" OR "interactive game*" OR "rehabilitation game*") AND ("Phantom Limb Pain"[Mesh] OR "phantom limb pain" OR PLP OR pain OR rehabilitation OR "motor recovery" OR "functional recovery" OR "prosthesis control" OR "motor control" OR embodiment OR "user experience" OR usability OR engagement))
Scopus	Elsevier	(TITLE-ABS-KEY(amputat* OR "limb loss" OR "upper limb amputation" OR "lower limb amputation")) AND (TITLE-ABS-KEY("virtual reality" OR "augmented reality" OR "mixed reality" OR "extended reality" OR VR OR AR OR MR OR XR)) AND (TITLE-ABS-KEY("serious game*" OR exergam* OR "game-based" OR gamified OR "interactive game*" OR "rehabilitation game*")) AND (TITLE-ABS-KEY("phantom limb pain" OR PLP OR pain OR rehabilitation OR "motor recovery" OR "functional recovery" OR "prosthesis control" OR "motor control" OR embodiment OR "user experience" OR usability OR engagement))
Web of Science	Clarivate	TS=(amputat* OR "limb loss" OR "upper limb amputation" OR "lower limb amputation") AND TS=("virtual reality" OR "augmented reality" OR "mixed reality" OR "extended reality" OR VR OR AR OR MR OR XR) AND TS=("serious game*" OR exergam* OR "game-based" OR gamified OR "interactive game*" OR "rehabilitation game*") AND TS=("phantom limb pain" OR PLP OR pain OR rehabilitation OR "motor recovery" OR "functional recovery" OR "prosthesis control" OR "motor control" OR embodiment OR "user experience" OR usability OR engagement)
IEEE Xplore	IEEE	(("All Metadata":amputat* OR "limb loss" OR "upper limb amputation" OR "lower limb amputation") AND ("All Metadata":"virtual reality" OR "augmented reality" OR "mixed reality" OR "extended reality" OR VR OR AR OR MR OR XR) AND ("All Metadata":"serious game*" OR exergam* OR "game-based" OR gamified OR "interactive game*" OR "rehabilitation game*") AND ("All Metadata":"phantom limb pain" OR PLP OR pain OR rehabilitation OR "motor recovery" OR "functional recovery" OR "prosthesis control" OR "motor control" OR embodiment OR "user experience" OR usability OR engagement))

Table SII. Summary of included studies on extended reality (XR) serious games for amputation rehabilitation and phantom limb pain management. For each study, the table reports the amputation level, sample size, XR modality, game type and title, task category, game mechanics, input control method, feedback modality, and principal reported outcomes. Abbreviations are presented in the footnote.

Study	Year	Country	Amputation	Sample Size Information	XR Modality	Game Type	Game Title / Example	Task Category	Game Mechanics	Input Control	Feedback Type	Reported Results
Lee et al. [1]	2026	USA (Johns Hopkins University School of Medicine / Johns Hopkins University)	Upper limb transradial intact-limb (IL) group 5M:1F, age mean: 26.33; Upper-limb-loss (ULL) group 2F, age: 71 years and 58 years	8 participants (6 able-bodied, 2 transradial amputees)	XR	Serious training game	Block Builder; Pasta Box Task; Cup Transfer Task	Prosthesis control training	Reach–grasp–transport–release tasks	EMG myoelectric control	Visual task feedback	Four 45-min XR training sessions in 6 IL and 2 ULL participants. Pasta Box Task completion rose from 20% to 100% in ULL; success rate 86.1±5.8% → 98.5±3.7% (IL) and 10%/0% → 90.9–100% (ULL). Iteration time dropped 19.4 → 15.8 s overall ($p < 0.001$). SUS 81.9 ± 10.0 (excellent).

Study	Year	Country	Amputation	Sample Size Information	XR Modality	Game Type	Game Title / Example	Task Category	Game Mechanics	Input Control	Feedback Type	Reported Results
Prahm et al. [2]	2025	Germany (University of Tübingen, BG Klinik)	Upper limb transradial/transcarpal Able-bodied (n = 10): 7M, 3F, age mean: 29.6 years Amputees (n = 8): 6M, 2F, age mean: 45.1 years	18 participants (10 able-bodied, 8 amputees)	Mixed Reality	Exploratory gamified therapy	PhantomAR environments	Phantom limb exploration	Curiosity-driven interaction with virtual objects	EMG threshold control	Visual + haptic vibration	Single-session mixed-reality mirror therapy in 8 transradial amputees with PLP + 10 able-bodied. PLP NRS fell ~58% (median 5 → 2, $p < 0.001$); SF-MPQ PRI dropped ~45% ($p < 0.001$). Residual-limb skin temperature rose 31.9 → 32.9 °C ($p < 0.01$) but did not correlate with PLP ($r = -0.08$, $p = 0.83$). SUS 90.8 (SD 6.88); GEQ immersion 4.46 (0.08), positive affect 4.97 (0.05). Tentacle

Study	Year	Country	Amputation	Sample Size Information	XR Modality	Game Type	Game Title / Example	Task Category	Game Mechanics	Input Control	Feedback Type	Reported Results
												overlay: mixed ownership, high agency.
Rozevink et al. [3]	2025	Netherlands (University of Groningen, University Medical Center Groningen – Department of Human Movement Sciences and Department of Rehabilitation Medicine)	Upper limb (wrist disarticulation / transradial) Prosthesis users (n = 11): 8M, 3F, age mean: 58.6 years; Therapists (n = 11): 1M, 10F, age mean: 49.3 years	Prosthesis users 11 analyzed; therapists 11	VR	ADL simulation game	Coffee-making task	ADL prosthesis training	Goal-based task completion with reward coins and progressive levels	Surface EMG pattern recognition + controller	Visual EMG activation feedback	Cross-sectional evaluation of a myoelectric-control VRE in 11 transradial prosthesis users and 11 therapists. SUS 82.5 [12.5] (users) and 75.0 [20.0] (therapists); no between-group difference. IMI subscales high (interest/enjoyment, importance, usefulness all > 6.4/7);

Study	Year	Country	Amputation	Sample Size Information	XR Modality	Game Type	Game Title / Example	Task Category	Game Mechanics	Input Control	Feedback Type	Reported Results
												perceived competence significantly lower ($p < 0.05$). PROS-TLX workload 23.0 [25.9] vs. 38.3 [16]; therapists scored significantly higher on visual attention ($p = 0.040$) and conscious processing ($p = 0.008$). All therapists would consider implementing the VRE in rehabilitation.

Study	Year	Country	Amputation	Sample Size Information	XR Modality	Game Type	Game Title / Example	Task Category	Game Mechanics	Input Control	Feedback Type	Reported Results
Jabbar et al. [4]	2024	Iraq (Baghdad Physical Rehabilitation Center and Sader Al Qanat Physical Rehabilitation Center)	Lower limb transtibial 100% male (n=22; only male participants completed the protocol) Age mean: Control 48.73 years ; EG 48.91 years	22 newly fitted transtibial amputees	Non-immersive VR	Exergame	Kinect Adventures (Reflex Ridge; River Rush); Kinect Sports (boxing)	Balance and gait rehabilitation	Sports and movement challenges	Controller-free body motion tracking (Kinect)	Not reported	Both (exergame and control) groups improved significantly on 2MWT, TUG, and AMPPRO (*p* <0.001). TUG time improved significantly more in the EG group vs control after 4 weeks (*p* =0.04, effect size = 0.53). No significant between-group difference on 2MWT, AMPPRO, or PCI.
Şahan and Erbahçeci [5]	2023	Turkey (University of Health Science Turkey, Gülhane Faculty of Physiother	Lower limb transtibial All male (n = 20) Age mean: mean 38.10 years	26 randomized; 20 analyzed	VR	Exergame	Sports and movement simulation games	Balance and gait rehabilitation	Sports-based movement tasks	Full-body motion capture (Kinect)	Not reported	Within-group gains in both arms (*p* < 0.05); no overall between-group difference.

Study	Year	Country	Amputation	Sample Size Information	XR Modality	Game Type	Game Title / Example	Task Category	Game Mechanics	Input Control	Feedback Type	Reported Results
		apy and Rehabilitation (Ankara))										6MWT: VR 227.65 → 263.92m; PT 228.60 → 387.90 m. Prosthetic-limb balance differed between groups at wk 3-4 (*p* = 0.029, 0.028). Cadence: VR 83 → 121; PT 50 → 62.
Annapureddy et al. [6]	2023	USA (The University of Texas Southwestern Medical School)	Lower limb amputation (n = 11), 10 M , 1 F age ranges: 35–40	11 Lower Limb Amputees	Mixed Reality	Therapeutic exergame	Bubble Burst; Pedal; Piano	Lower limb movement training	Repetitive movement tasks	Camera-based limb tracking	Visual feedback	MPQ "current pain intensity" 1.75 → 1.125/5 (*p* = 0.011; ≈ 35 % ↓); constrictive subclass 3.75 → 2.0 (*p* = 0.049); evaluative subclass 2.125 → 1.625 (*p* = 0.033). PSFS 4.28 → 6.22/10 (*p* = 0.006; 45 %

Study	Year	Country	Amputation	Sample Size Information	XR Modality	Game Type	Game Title / Example	Task Category	Game Mechanics	Input Control	Feedback Type	Reported Results
Yoshimura et al. [7]	2023	Japan (Faculty of Rehabilitation, Kawasaki University of Medical Welfar)	Upper limb (digit amputation) 1 female participant, age: 42 years	1 participant	VR	Motor training game	Catching rolling ball	Object manipulation training	Catching moving objects	Controller input	Visual + vibration	↑). BPI interference 4.75 → 1.39/10 (p = 0.037). Weekly pre-exercise pain 3.375 → 2.51 at Week 4 (p = 0.019). Enrolment 73.3 %; completion 72.7 %; no adverse events. SF-MPQ-2: 147 → 128 → 93 → 100/220 across Periods A, B, A', B'. Gnawing, piercing, punishing-cruel, tiring-exhausting qualities resolved (8 → 0); throbbing/stabbing/sharp rebounded in B'. 24-h amputated-

Study	Year	Country	Amputation	Sample Size Information	XR Modality	Game Type	Game Title / Example	Task Category	Game Mechanics	Input Control	Feedback Type	Reported Results
												limb activity 1,076,658 → 2,542,317 vector-magnitude units (reached parity with intact limb). Immersion VAS 95.2 ± 9.6 (B); 100 ± 0.0 (B'). Agency 6.4 ± 0.8 → 7.0 ± 0.0 (Likert 1–7).
El Raghibi et al. [8]	2023	Belgium (UCLouvain)	Upper limb transradial 6 M / 7 F (n=13; 12 able-bodied + 1 male ULA participant, S13) Able-bodied (S01–S12) age range: 21–26 years; ULA participant (S13) age: 27 years	13 participants (12 able-bodied + 1 amputee)	VR	Prosthesis training simulation	Grip selection tasks	Prosthetic hand control	Object manipulation tasks	Surface EMG control	Visual + auditory feedback	Task duration decreased significantly across sessions for Tasks 1 and 2a (Prosthesis 1 scored significantly higher on usability than both Prosthesis 2 and Prosthesis 3).

Study	Year	Country	Amputation	Sample Size Information	XR Modality	Game Type	Game Title / Example	Task Category	Game Mechanics	Input Control	Feedback Type	Reported Results
Annaswamy et al. [9]	2022	USA (Institutes: Penn State Health Milton S. Hershey Medical Center)	Lower limb 6M, 1F (n = 7 enrolled Age mean: 64 years	7 enrolled; 4 completed	Mixed Reality mirror therapy	Therapeutic exercises	Exergames	Lower limb exercises	Exergame interaction	Camera-based limb mirroring	Visual feedback	Recruitment 78%; retention 57% (4/7). PSFS improved in 2/3 (overall 4.625 → 5.75); no clear trend on NRS or MPQ pain. All completers reported satisfaction and temporary pain relief.
Shim et al. [10]	2022	Republic of Korea (Kwangwoon University / Seoul Asan Medical Center)	Lower limb Not reported (usability test with 8 public + 7 amputees; gender distribution not specified) Age range: 30–60 years	Lower limb amputee patients	XR (MR/VR)	Game-based XR rehabilitation	Not reported	Motor rehabilitation tasks	Movement scoring tasks	EMG signal control	Score-based feedback	Deep learning model for EMG motion detection achieved 92.43% accuracy (loss 0.405%, inference time 0.98ms). Usability evaluation (*t*-test) showed significant differences between public and

Study	Year	Country	Amputation	Sample Size Information	XR Modality	Game Type	Game Title / Example	Task Category	Game Mechanics	Input Control	Feedback Type	Reported Results
Pérez et al. [11]	2022	Canada (University of British Columbia)	Lower limb Usability sample (n = 6): 3F physiotherapists, 2M physiotherapists, 1M lower-limb amputee (51 years); health-professional survey (n = 13), age mean: 30-37 years: not	6 participants	VR	VR exergame rehabilitation	Exergame (Exogames)	Lower limb rehabilitation exercises	Exergame	Body movement via robot sensors	Score and immersive feedback	amputation groups on items 2.5 (*p* =0.025), 2.6 (*p* = 0.036), 3.5 (*P* = 0.022), and 3.7 (*p* = 0.040). Motivation rated highly across both groups; older users reported difficulty adapting to HoloLens 2. SUS 69 (66, 79); PACES 110/126 (108, 112); GEQ immersion 4 (3, 5), positive affect 4 (2, 4), tension 1 (1, 2). Expert survey (n = 13): 66.7% endorsed physical rehab fit, 83.3% wellness,

Study	Year	Country	Amputation	Sample Size Information	XR Modality	Game Type	Game Title / Example	Task Category	Game Mechanics	Input Control	Feedback Type	Reported Results
			specified by sex									76.9% interface/reporting.
Hashim et al. [12]	2021	Malaysia (Universiti Malaya)	Upper limb transradial Not explicitly reported (both male and female participants referenced; mean age 26.3 ± 4.47 years; n=10, 5 amputees + 5 able-bodied) Age mean: 26.3	10 participants (5 amputees, 5 controls)	Video-game / VR training	Commercial video games	Fruit Ninja; Crate Whacker; Race the Sun; Kaiju Carnage	Motor coordination training	Gesture interaction tasks	EMG gesture control	Game rewards and interaction	Significant positive correlation between training period and BBT score (r_s=0.95, p <0.001). MVC values improved from pre-training to follow-up for all participants; 9/10 showed MVC improvement at follow-up vs baseline. IMI showed high scores for interest/enjoyment, perceived competence, choice, and value/usefuln

Study	Year	Country	Amputation	Sample Size Information	XR Modality	Game Type	Game Title / Example	Task Category	Game Mechanics	Input Control	Feedback Type	Reported Results
												ess; low for pressure/tension.
Abbas et al. [13]	2021	Lebanon (Physical Therapy Department, Faculty of Health Sciences, Beirut Arab University)	Lower limb unilateral traumatic (n = 32) Control: 14 M, 2 F, Age mean: 27.6 years VR Group: 15 M, 1 F, age mean: 27.6 years	32 participants	VR	Rehabilitation exergames	Football; obstacle crossing; tennis; multidirectional movement	Balance and gait	Sports and obstacle tasks	Body motion tracking	Score feedback	Between-group (VR + exercise vs. exercise): DGI 17.81 → 22.75 (p = 0.0001); TUG 11.11 → 8.05 s (p = 0.001); BBS 43.19 → 51.38 (p = 0.035); 6MWT 166.19 → 262.63 m (within-group p = 0.0003; between-group p = 0.091, ns).

Study	Year	Country	Amputation	Sample Size Information	XR Modality	Game Type	Game Title / Example	Task Category	Game Mechanics	Input Control	Feedback Type	Reported Results
Ambron et al. [14]	2021	USA (University of Pennsylvania / Moss Rehabilitation / University of Delaware)	Lower limb transtibial 4 M / 3 F (n=7) Individual ages: 46, 57, 54, 52, 53, 48, 50 years	8 enrolled; 7 analyzed	VR	Custom VR therapy games	Custom VR games	Phantom limb motor control	Limb movement tasks	Motion tracking	Game feedback	PLP decreased 28% after distractor treatment and 39.6% after limb treatment. Reduction after limb treatment survived Bonferroni correction ($p = 0.015$); distractor reduction did not ($p = 0.046$). No significant difference between the two treatments. BPI pain interference decreased more with limb treatment than distractor (28% vs 15%, p =0 .03). Trends

Study	Year	Country	Amputation	Sample Size Information	XR Modality	Game Type	Game Title / Example	Task Category	Game Mechanics	Input Control	Feedback Type	Reported Results
												toward reduced depression and anxiety after limb treatment.
Thøgersen et al. [15]	2020	Denmark (Center for Neuroplasticity and Pain (CNAP), Department of Health Science and Technology, Faculty of Medicine, Aalborg University	Upper limb amputees (n = 8), 5 M, 3 F Age mean ≈ 48.4 years; range 33–75	8 participants (7 analyzed)	Augmented Reality	Rehabilitation serious games	Pick-and-place; imitation; sorting	Phantom motor execution	Object sorting and imitation	EMG pattern recognition	Game scoreboards and task feedback	McGill SF PLP ↓ 52 % per session (mean Δ = −1.884; p = 0.032; d = 1.135). Diary NRS 1.94 → 1.31 (32 % ↓); responders (n = 6) ↓ 41 % (p = 0.022; d = 1.334). fMRI lip-pursing: significant ↓ S1 activity contralateral to amputation (BA 3b; p = 0.011); cortical lip representation shifted 4.62 mm (SD 3.87) toward healthy-side location (p =

Study	Year	Country	Amputation	Sample Size Information	XR Modality	Game Type	Game Title / Example	Task Category	Game Mechanics	Input Control	Feedback Type	Reported Results
Kulkarni et al. [16]	2020	UK (North of England Specialised Ability Centre; University of Manchester)	Upper limb amputees 7 M / 2 F among 9 completers (11 recruited: 7 M / 4 F) Age range: 46–80 years	11 enrolled; 9 analyzed	VR	Game-like therapy task	Ball interaction task	Motor control	Object interaction	Limb motion tracking	Visual feedback	0.056). Embodiment–PLP r = −0.78 (p = 0.040); agency–PLP r = −0.75 (p = 0.055); embodiment/agency–telescoping both r = 0.90. Mean PLP NRS dropped from 6.11 to 3.56 after three VR sessions (not statistically significant, t=2.1, df = 8, p = 0.07). No significant difference in number of PLP episodes (p = 0.18) or duration (p = 0.13). At 1-year follow-up, mean NRS was 5.56 (p = 0.21). 6/9 reported reduced NRS

Study	Year	Country	Amputation	Sample Size Information	XR Modality	Game Type	Game Title / Example	Task Category	Game Mechanics	Input Control	Feedback Type	Reported Results
												post-trial; qualitative reports noted hand unclenching and requests for longer treatment.
Lendaro et al. [17]	2020	Sweden (Chalmers University of Technology, Biomechatronics and Neurorehabilitation Laboratory)	Upper + lower limb amputees 3M, 1F (n = 4) Ages: S1: 77 years; S2: 56 years; S3: 72 years; S4: 28 years	4 participants	AR/VR	Serious rehabilitation games	Racing game; TAC test	Motor execution training	Target control tasks	EMG pattern recognition	Real-time feedback	2-month in-home use of phantom motor execution with AR/VR in 2 upper- and 2 lower-limb amputees. Self-reported PLP (SF-MPQ PRI) remained low and stable throughout; adherence to the recommended 8 sessions/month peaked in the first weeks then declined, with ad-hoc

Study	Year	Country	Amputation	Sample Size Information	XR Modality	Game Type	Game Title / Example	Task Category	Game Mechanics	Input Control	Feedback Type	Reported Results
												resumption driven by pain recurrence. Session durations generally fell below the advised 90 min. Two distinct user types emerged (goal-oriented vs. experiential); main adherence barriers were time and single-use electrode availability.
Tao et al. [18]	2020	Canada (University of British Columbia (Vancouver))	Lower limb amputees 16% female overall (n = 62); experimental 7F:27M, control 3F:25M Age mean: 66.4 years	64 participants	Non-immersive VR	Exergame	Nintendo Wii Fit games	Balance and walking	Balance board exercises	Weight shifting	Score feedback	Unsupervised/supervised session-count ratios below parity in both groups (experimental 0.25 vs. control 0.18; $p = 0.92$). Session-time ratios

Study	Year	Country	Amputation	Sample Size Information	XR Modality	Game Type	Game Title / Example	Task Category	Game Mechanics	Input Control	Feedback Type	Reported Results
												favoured exp arm (1.12 vs. 0.76; p = 0.027). Supervised adherence 56% (experimental) / 71% (control).
Ambron et al. [19]	2018	USA (University of Pennsylvania / Moss Rehabilitation)	Lower limb transtibial Not reported (2 case subjects described only as "late-middle-aged" and "middle-aged"; gender not specified)	2 participants	VR	Custom VR rehabilitation games	Custom VR games	Motor rehabilitation	Interactive limb movement	Motion sensors	Game feedback	Subject 1: post-session pain reduced 100% in both sessions; pre-session pain decreased 22% from session 1 to 2. Subject 2: post-session pain reduced by an average of 93.7%; pre-session pain decreased 67% from session 1 to session 4. Five of six post-session pain scores were 0/10.

Study	Year	Country	Amputation	Sample Size Information	XR Modality	Game Type	Game Title / Example	Task Category	Game Mechanics	Input Control	Feedback Type	Reported Results
												SUS ratings: 3 of 4 scores above 50/100.
Imam et al. [20]	2017	Canada (University of British Columbia)	Lower limb amputees (n = 14), 12 M, 2 F, Median age: 62 years	28 randomized; 24 analyzed	Non-immersive VR	Exergame	Wii Fit games	Walking and balance	Balance training games	Weight shifting	Game feedback	2MWT Cohen's d = 0.5 (end of treatment) and 0.6 (3-week retention) ITT; d = 0.9 and 1.2 per protocol. Mean between-group walking gain: 6.5 m / 7.4 m (ITT); 11.3 m / 12.1 m (per protocol). SPPB d = 0.7 (end) and 0.8 (retention). Adherence 83.4 %; perceived benefit 38.9/45 (SD 6.8). Sample required for

Study	Year	Country	Amputation	Sample Size Information	XR Modality	Game Type	Game Title / Example	Task Category	Game Mechanics	Input Control	Feedback Type	Reported Results
												definitive RCT = 72 (36/arm).
Chau et al. [21]	2017	USA (Loma Linda University Health, California)	Upper limb amputee 1 M (49 years)	1 participant	VR	ADL + game tasks	Kitchen interaction tasks; VR games	ADL rehabilitation	Task completion	EMG control	Interactive environment	Statistically significant decreases per session: VAS pain scores decreased 55% (p = 0.0143), SF-MPQ 60% (p = 0.023), Wong-Baker FACES 90% (p = 0.0024). Patient reported pain relief lasting ~24 hours and progressively longer across sessions; phantom hand posture gradually relaxed from clenched fist. Continued benefit at 6-week follow-up.

Study	Year	Country	Amputation	Sample Size Information	XR Modality	Game Type	Game Title / Example	Task Category	Game Mechanics	Input Control	Feedback Type	Reported Results
Ortiz-Catalan et al. [22]	2016	Sweden (Department of Signals and Systems, Chalmers University of Technology)	Mostly upper limb amputees (n = 14), gender distribution not specified. Age: 72 years	14 participants	AR/VR	Serious rehabilitation games	Target tasks and VR environments	Phantom motor execution	Task-based control	EMG pattern recognition	Visual and gaming feedback	PLP ↓ 47 % WPD (p = 0.001), 32 % NRS (p = 0.007), 51 % PRI (p = 0.0001). ADL intrusion NRS ↓ 43 % (p = 0.004); sleep intrusion ↓ 61 % (p = 0.001). 2/4 medicated patients cut intake by 81 % and 33 %. "Stabbing" and "tiring-exhausting" qualities significantly less prevalent (p = 0.016). Gains maintained at 6-month follow-up (PRI decayed only 2%, 6%, 24% at 1, 3, 6 months).

Study	Year	Country	Amputation	Sample Size Information	XR Modality	Game Type	Game Title / Example	Task Category	Game Mechanics	Input Control	Feedback Type	Reported Results
Ortiz-Catalan et al. [23]	2014	Sweden (Chalmers University of Technology / Sahlgrenska University Hospital)	Upper limb amputee 1 M (72 years)	1 participant	Augmented Reality	Rehabilitation gaming therapy	Racing game	Phantom limb motor control	Racing interaction	EMG pattern recognition	Game feedback	Sustained pain level gradually reduced over 18 weeks, with complete pain-free periods emerging after ~10 weeks. Phantom hand posture evolved from permanently clenched fist (48 years) to a mid-open relaxed position after 6 sessions. Patient regained voluntary phantom limb motion control; telescopic effect restored phantom hand to anatomically correct distance.

Study	Year	Country	Amputation	Sample Size Information	XR Modality	Game Type	Game Title / Example	Task Category	Game Mechanics	Input Control	Feedback Type	Reported Results
Imam et al.[24]	2013	Canada (University of British Columbia)	Lower limb amputees 5M, 1F (n = 6 completers) Age mean: 48.5 years	6 participants	Non-immersive VR	Exergame	Wii Fit exercises	Balance and gait	Exercise games	Weight shifting	Game feedback	Motion test completion rate 98.0% (±2), real-time accuracy 75% (±4.2) after week 15. Adherence 80%; SFQ-M 35/50. Statistical improvement on 2MWT in 5/6 (final median 166m), SPPB in 4/6, ABC in 3/6, L-test in 2/6. No adverse events.

Abbreviations: 2MWT, 2-Minute Walk Test; 6MWT, 6-Minute Walk Test; ABC, Activities-specific Balance Confidence Scale; ADL, Activities of Daily Living; AMPPRO, Amputee Mobility Predictor with Prosthesis; AR, Augmented Reality; BA, Brodmann Area; BBS, Berg Balance Scale; BBT, Box and Block Test; BPI, Brief Pain Inventory; *d*, Cohen's *d* (effect size); *df*, degrees of freedom; DGI, Dynamic Gait Index; EG, Exergame Group; EMG, Electromyography; fMRI, functional Magnetic Resonance Imaging; GEQ, Game Experience Questionnaire; IL, Intact Limb; IMI, Intrinsic Motivation Inventory; ITT, Intention-to-Treat; MPQ, McGill Pain Questionnaire; MR, Mixed Reality; MVC, Maximal Voluntary Contraction; NRS, Numeric Rating Scale; *ns*, not significant; PACES, Physical Activity Enjoyment Scale; PCI, Physiological Cost Index; PLP, Phantom Limb Pain; PRI, Pain Rating Index; PSFS, Patient-Specific Functional Scale; PT, Physical Therapy; *r*, Pearson correlation coefficient; S1, primary somatosensory cortex; SD, Standard Deviation; sEMG, surface Electromyography; SF-MPQ, Short-Form McGill Pain Questionnaire; SF-MPQ-2, Short-Form McGill Pain Questionnaire–2; SFQ-M, Satisfaction with Function Questionnaire–

Modified; SPPB, Short Physical Performance Battery; SUS, System Usability Scale; TAC, Target Achievement Control; TUG, Timed Up and Go test; ULL, Upper-Limb Loss; VAS, Visual Analogue Scale; VR, Virtual Reality; WPD, Weighted Pain Distribution; XR, Extended Reality.

References

[1] Lee I, Hunt CL, Thakor N V, Kaliki RR. Design and Evaluation of User-Centered Extended Reality Myoelectric Prosthesis Training Tool. IEEE TRANSACTIONS ON NEURAL SYSTEMS AND REHABILITATION ENGINEERING 2026;34:2026. https://doi.org/10.57922/MEC.2483.

[2] Prahm C, Eckstein K, Bressler M, Wang Z, Li X, Suzuki T, et al. PhantomAR: gamified mixed reality system for alleviating phantom limb pain in upper limb amputees—design, implementation, and clinical usability evaluation. J Neuroeng Rehabil 2025;22. https://doi.org/10.1186/s12984-025-01554-7.

[3] Rozevink SG, Maas B, Murgia A, Bongers RM, van der Sluis CK. Therapists' and prosthesis users' assessments of a virtual reality environment designed for upper limb prosthesis control training. Journal of Hand Therapy 2025. https://doi.org/10.1016/j.jht.2025.01.004.

[4] Jabbar MAH, Ashnagar Z, Hadian MR, Jalaie S, Moghadam ST. Investigating the Effects of Exergame Training on Functional Activities Among Newly-Fitted Patients with Unilateral Transtibial Amputation: A Preliminary Study. Journal of Modern Rehabilitation 2024;18:371–82. https://doi.org/10.18502/jmr.v18i3.16424.

[5] Sahan TY, Erbahçeci F. Effects of Virtual Reality on Transtibial Amputation Rehabilitation Outcomes: A Randomized Study. Games Health J 2023;12:459–67. https://doi.org/10.1089/g4h.2023.0052.

[6] Annapureddy D, Annaswamy TM, Raval G, Chung YY, Prabhakaran B. A novel mixed reality system to manage phantom pain in-home: results of a pilot clinical trial. Frontiers in Pain Research 2023;4. https://doi.org/10.3389/fpain.2023.1183954.

[7] Yoshimura M, Kurumadani H, Hirata J, Senoo K, Hanayama K, Sunagawa T, et al. Case Report: Virtual reality training for phantom limb pain after amputation. Front Hum Neurosci 2023;17. https://doi.org/10.3389/fnhum.2023.1246865.

[8] Raghibi L El, Muhoza AP, Evrard J, Ghazi H, van Oldeneel tot Oldenzeel G, Sonneville V, et al. Virtual reality can mediate the learning phase of upper limb prostheses supporting a better-informed selection process. Journal on Multimodal User Interfaces 2023;17:33–46. https://doi.org/10.1007/s12193-022-00400-7.

[9] Annaswamy TM, Bahirat K, Raval G, Chung YY, Pham T, Prabhakaran B. Clinical feasibility and preliminary outcomes of a novel mixed reality system to manage phantom pain: a pilot study. Pilot Feasibility Stud 2022;8. https://doi.org/10.1186/s40814-022-01187-w.

[10] Shim W, Kim H, Lim G, Lee S, Kim H, Hwang J, et al. Implementation of the XR Rehabilitation Simulation System for the Utilization of Rehabilitation with Robotic Prosthetic Leg. Applied Sciences (Switzerland) 2022;12. https://doi.org/10.3390/app122412659.

[11] Pérez VZ, Yepes JC, Vargas JF, Franco JC, Escobar NI, Betancur L, et al. Virtual Reality Game for Physical and Emotional Rehabilitation of Landmine Victims. Sensors 2022;22. https://doi.org/10.3390/s22155602.

[12] Hashim NA, Abd Razak NA, Gholizadeh H, Abu Osman NA. Video game–based rehabilitation approach for individuals who have undergone upper limb amputation: Case-control study. JMIR Serious Games 2021;9. https://doi.org/10.2196/17017.

[13] Abbas RL, Cooreman D, Al Sultan H, El Nayal M, Saab IM, El Khatib A. The Effect of Adding Virtual Reality Training on Traditional Exercise Program on Balance and Gait in Unilateral, Traumatic Lower Limb Amputee. Games Health J 2021;10:50–6. https://doi.org/10.1089/g4h.2020.0028.

[14] Ambron E, Buxbaum LJ, Miller A, Stoll H, Kuchenbecker KJ, Coslett HB. Virtual Reality Treatment Displaying the Missing Leg Improves Phantom Limb Pain: A Small Clinical Trial. Neurorehabil Neural Repair 2021;35:1100–11. https://doi.org/10.1177/15459683211054164.

[15] Thøgersen M, Andoh J, Milde C, Graven-Nielsen T, Flor H, Petrini L. Individualized Augmented Reality Training Reduces Phantom Pain and Cortical Reorganization in Amputees: A Proof of Concept Study. Journal of Pain 2020;21:1257–69. https://doi.org/10.1016/j.jpain.2020.06.002.

[16] Kulkarni J, Pettifer S, Turner S, Richardson C. An investigation into the effects of a virtual reality system on phantom limb pain: a pilot study. Br J Pain 2020;14:92–7. https://doi.org/10.1177/2049463719859913.

[17] Lendaro E, Middleton A, Brown S, Ortiz-Catalan M. Out of the clinic, into the home: The in-home use of phantom motor execution aided by machine learning and augmented reality for the treatment of phantom limb pain. J Pain Res 2020;13:195–209. https://doi.org/10.2147/JPR.S220160.

[18] Tao G, Miller WC, Eng JJ, Lindstrom H, Imam B, Payne M. Self-directed usage of an in-home exergame after a supervised telerehabilitation training program for older adults with lower-limb amputation. Prosthet Orthot Int 2020;44:52–9. https://doi.org/10.1177/0309364620906272.

[19] Ambron E, Miller A, Kuchenbecker KJ, Buxbaum LJ, Coslett HB. Immersive low-cost virtual reality treatment for phantom limb pain: Evidence from two cases. Front Neurol 2018;9. https://doi.org/10.3389/fneur.2018.00067.

[20] Imam B, Miller WC, Finlayson H, Eng JJ, Jarus T. A randomized controlled trial to evaluate the feasibility of the Wii Fit for improving walking in older adults with lower limb amputation. Clin Rehabil 2017;31:82–92. https://doi.org/10.1177/0269215515623601.

[21] Chau B, Phelan I, Ta P, Humbert S, Hata J, Tran D. Immersive virtual reality therapy with myoelectric control for treatment-resistant phantom limb pain: case report. Innov Clin Neurosci 2017;14:3.

[22] Ortiz-Catalan M, Guðmundsdóttir RA, Kristoffersen MB, Zepeda-Echavarria A, Caine-Winterberger K, Kulbacka-Ortiz K, et al. Phantom motor execution facilitated by machine learning and augmented reality as treatment for phantom limb pain: a single group, clinical trial in patients with chronic intractable phantom limb pain. The Lancet 2016;388:2885–94.

[23] Ortiz-Catalan M, Sander N, Kristoffersen MB, Håkansson B, Brånemark R. Treatment of phantom limb pain (PLP) based on augmented reality and gaming controlled by myoelectric pattern recognition: A case study of a chronic PLP patient. Front Neurosci 2014. https://doi.org/10.3389/fnins.2014.00024.

[24] Imam B, Miller WC, McLaren L, Chapman P, Finlayson H. Feasibility of the Nintendo WiiFit™ for improving walking in individuals with a lower limb amputation. SAGE Open Med 2013;1. https://doi.org/10.1177/2050312113497942.

Table SIII. MMAT quality appraisal of included studies.

Study	Design	S1	S2	C1	C2	C3	C4	C5	Score /5
Rozevink (2025)	Qual	Yes	Yes	Yes	Yes	U	U	U	2/5
Lee (2026)	QD	Yes	Yes	No	Yes	U	U	U	1/5
Prahm (2025)	QD	Yes	Yes	No	Yes	U	Yes	U	2/5
Annapureddy (2023)	QD	Yes	Yes	U	Yes	U	U	U	1/5
El Raghibi (2023)	QD	Yes	Yes	No	Yes	U	U	U	1/5
Shim (2022)	QD	U	U	No	No	No	No	No	0/5
Perez (2022)	QD	Yes	Yes	U	Yes	U	U	U	1/5
Hashim (2021)	QD	Yes	Yes	U	Yes	Yes	U	U	2/5
Annaswamy (2022)	QD	Yes	Yes	U	No	No	U	No	0/5
Lendaro (2020)	QD	Yes	Yes	No	Yes	Yes	U	U	1/5
Ambron (2018)	QD	Yes	Yes	No	Yes	Yes	U	U	1/5
Ortiz-Catalan (2014)	QD	Yes	Yes	No	Yes	Yes	U	U	1/5
Imam (2013)	QD	Yes	Yes	U	Yes	Yes	U	U	2/5
Yoshimura (2023)	Case	Yes	Yes	Yes	Yes	U	U	Yes	3/5
Chau (2017)	Case	Yes	Yes	Yes	Yes	U	U	Yes	3/5
Jabbar (2024)	QNR	Yes	Yes	Yes	Yes	U	Yes	No	3/5
Thogersen (2020)	QNR	Yes	Yes	U	Yes	No	Yes	U	3/5
Ambron (2021)	QNR	Yes	Yes	U	Yes	No	U	Yes	3/5
Kulkarni (2020)	QNR	Yes	Yes	U	Yes	No	U	No	1/5
Ortiz-Catalan (2016)	QNR	Yes	Yes	Yes	Yes	No	Yes	Yes	4/5

Study	Design	S1	S2	C1	C2	C3	C4	C5	Score /5
Sahan (2023)	RU	**Yes**	**Yes**	**Yes**	**No**	**No**	U	U	**1/5**
Abbas (2021)	RU	**Yes**	**Yes**	**Yes**	U	**Yes**	**Yes**	**Yes**	**4/5**
Tao (2020)	RU	**Yes**	**Yes**	**Yes**	U	**Yes**	U	U	**2/5**
Imam (2017)	RU	**Yes**	**Yes**	**Yes**	**Yes**	**Yes**	**Yes**	**Yes**	**5/5**

Design abbreviations: Qual = Qualitative; QD = Quantitative Descriptive; QNR = Quantitative Non-Randomised; RCT = Randomised Controlled Trial; Case = Case Report. Screening questions: S1 = clear research question present; S2 = collected data appropriate to address the research question. Type-specific criteria (C1–C5) vary by design category. U = Unsure. Score reflects the number of type-specific criteria rated Yes *out of five.*

Table SIV. GRADE summary of findings for the included studies.

Outcome	Studies (n)	Design	Risk of bias	Inconsistency	Indirectness	Imprecision	Certainty
Phantom limb pain reduction (NRS / VAS / SF-MPQ)	10	Non-randomised; case reports; descriptive	Very serious	Serious	Not serious	Very serious	Very Low
Functional outcomes – balance, gait & walking capacity (BBS, TUG, 2MWT, 6MWT)	9	RCT (n=2); non-randomised; descriptive	Serious	Not serious	Not serious	Serious	Low
Prosthesis control & training performance (task time, BBT, EMG accuracy)	9	Descriptive; non-randomised	Very serious	Serious	Serious	Very serious	Very Low
Usability & user experience (SUS, IMI, GEQ, PROS-TLX)	6	Qualitative; descriptive	Serious	Not serious	Not serious	Serious	Low
Embodiment & neurofunctional outcomes (ownership/agency, skin temperature, fMRI, accelerometry)	6	Non-randomised; descriptive; case report	Very serious	Undetected	Serious	Very serious	Very Low

Risk of bias, inconsistency, indirectness, and imprecision rated as Not serious, Serious, or Very serious. Undetected = insufficient studies to assess domain. Low = further research likely to change the estimate; Very Low = any estimate is very uncertain. NRS = Numerical Rating Scale; VAS = Visual Analogue Scale; SF-MPQ = Short-Form McGill Pain Questionnaire; BBS = Berg Balance Scale; TUG = Timed Up and Go; 2MWT/6MWT = 2- and 6-Minute Walk Tests; BBT = Box and Block Test; EMG = electromyography; SUS = System Usability Scale; IMI = Intrinsic Motivation Inventory; GEQ = Game Experience Questionnaire; fMRI = functional magnetic resonance imaging; RCT = randomised controlled trial.
